# New *Drosophila* Circadian Clock Mutants Affecting Temperature Compensation Induced by Targeted Mutagenesis of *Timeless*

**DOI:** 10.3389/fphys.2019.01442

**Published:** 2019-12-03

**Authors:** Samarjeet Singh, Astrid Giesecke, Milena Damulewicz, Silvie Fexova, Gabriella M. Mazzotta, Ralf Stanewsky, David Dolezel

**Affiliations:** ^1^Institute of Entomology, Biology Centre of Academy of Sciences of the Czech Republic, České Budějovice, Czechia; ^2^Faculty of Science, University of South Bohemia in České Budějovice, České Budějovice, Czechia; ^3^Institute of Neuro- and Behavioral Biology, Westfälische Wilhelms University, Münster, Germany; ^4^Department of Cell Biology and Imaging, Institute of Zoology and Biomedical Research, Jagiellonian University, Kraków, Poland; ^5^Department of Biology, University of Padua, Padua, Italy

**Keywords:** circadian clock, reverse genetics, screening, candidate genes, temperature compensation, CRISPR-CAS9, *Drosophila melanogaster*

## Abstract

*Drosophila melanogaster* has served as an excellent genetic model to decipher the molecular basis of the circadian clock. Two key proteins, PERIOD (PER) and TIMELESS (TIM), are particularly well explored and a number of various arrhythmic, slow, and fast clock mutants have been identified in classical genetic screens. Interestingly, the free running period (tau, *τ*) is influenced by temperature in some of these mutants, whereas *τ* is temperature-independent in other mutant lines as in wild-type flies. This, so-called “temperature compensation” ability is compromised in the mutant *timeless* allele *“ritsu”* (*tim*^*rit*^), and, as we show here, also in the *tim*^*blind*^ allele, mapping to the same region of TIM. To test if this region of TIM is indeed important for temperature compensation, we generated a collection of new mutants and mapped functional protein domains involved in the regulation of τ and in general clock function. We developed a protocol for targeted mutagenesis of specific gene regions utilizing the CRISPR/Cas9 technology, followed by behavioral screening. In this pilot study, we identified 20 new *timeless* mutant alleles with various impairments of temperature compensation. Molecular characterization revealed that the mutations included short in-frame insertions, deletions, or substitutions of a few amino acids resulting from the non-homologous end joining repair process. Our protocol is a fast and cost-efficient systematic approach for functional analysis of protein-coding genes and promoter analysis *in vivo*. Interestingly, several mutations with a strong temperature compensation defect map to one specific region of TIM. Although the exact mechanism of how these mutations affect TIM function is as yet unknown, our *in silico* analysis suggests they affect a putative nuclear export signal (NES) and phosphorylation sites of TIM. Immunostaining for PER was performed on two TIM mutants that display longer *τ* at 25°C and complete arrhythmicity at 28°C. Consistently with the behavioral phenotype, PER immunoreactivity was reduced in circadian clock neurons of flies exposed to elevated temperatures.

## Introduction

Circadian clocks orchestrate the physiology, metabolism, and behavior of living organisms to be optimally aligned to the periodic day and night changes in the environment. For that reason, circadian clocks “keep ticking” even under constant conditions with a free running period (*tau*, *τ*) close to 24 h. A crucial functional feature of circadian clocks is their ability to run with a comparable speed at a wide range of physiological temperatures, a phenomenon termed “temperature compensation.” From a mechanistic point of view, these biological oscillators are a series of interconnected biochemical reactions, which involve transcriptional and translational feedback loops. The exceptional genetic tools available in the fruit fly, *Drosophila melanogaster*, have enabled the identification and detailed analysis of the functional components of the circadian system and their interactions. Many excellent and detailed reviews are available on this topic (Hardin, 2011; Ozkaya and Rosato, 2012; Tataroglu and Emery, 2015; Tomioka and Matsumoto, 2015).

At the core of the fruit fly’s circadian clock, the transcription factors CLOCK (CLK) and CYCLE (CYC) drive the expression of genes with E-box motif(s) in the promoter region, including *period* (*per*) and *timeless* (*tim*). PER and TIM proteins slowly accumulate, dimerize in the cytoplasm, and later start to translocate to the cell nucleus, where they inhibit CLK–CYC mediated transcription (Darlington et al., 1998; Glossop et al., 1999). As a result of this negative feedback loop, *per* and *tim* mRNA is repressed, which consequently results in depletion of PER and TIM proteins, allowing the whole cycle to start again with a new round of CLK-CYC mediated transcription. Several kinases and phosphatases tightly regulate the stability of PER and TIM, fine-tuning the pace of the oscillator to roughly 24 h (Price et al., 1998; Martinek et al., 2001; Sathyanarayanan et al., 2004; Agrawal and Hardin, 2016). Additional interconnected transcription/translational feedback loops that contribute to the circadian system were described in *Drosophila* as well as other insects. The PER/TIM feedback loop model was established and further refined through a combination of immunocytochemistry (ICC) (Siwicki et al., 1988), time-course expression profiling (Hardin et al., 1990, 1992), protein biochemical approaches addressing phosphorylation (Edery et al., 1994; Chiu et al., 2011), glycosylation (Li et al., 2019), protein coexpression in *Drosophila* Schneider cell culture (Saez and Young, 1996; Nawathean and Rosbash, 2004; Meyer et al., 2006), and yeast two-hybrid experiments (Rutila et al., 1996). But the key starting point in the *per* and *tim* research was the identification of mutants in extensive genetic screens using either chemical mutagens (Konopka and Benzer, 1971; Konopka et al., 1994; Rothenfluh et al., 2000a), or P-element mobilization (Sehgal et al., 1994). Additionally, spontaneous clock mutations were recovered from wild populations (Matsumoto et al., 1999), or laboratory stocks (Hamblen et al., 1998). Importantly, not only null mutations were obtained, but also mutants with altered protein sequences resulting in faster or slower *τ* in both *per* (Konopka and Benzer, 1971; Konopka et al., 1994; Hamblen et al., 1998) and *tim* (Matsumoto et al., 1999; Rothenfluh et al., 2000a, b; Wülbeck et al., 2005) genes.

The protein–protein interaction between PER and TIM is a complex and dynamic event (Meyer et al., 2006), including PER homodimerization (Landskron et al., 2009), multiple sequential phosphorylations (Martinek et al., 2001; Ko et al., 2010; Chiu et al., 2011), dephosphorylations (Sathyanarayanan et al., 2004; Fang et al., 2007), and possibly additional posttranslational modifications (Li et al., 2019). A key feature of the negative feedback loop in *Drosophila* is the ∼ 6 h delay that exists between the cytoplasmic accumulation and nuclear translocation of PER and TIM. Both PER and TIM proteins contain a nuclear localization signal (NLS) and cytoplasmic localization domain (CLD) (Saez and Young, 1996). Transgenic flies with mutated TIM NLS have a slower *τ*, and even though PER and TIM reach high cytoplasmic levels, their nuclear translocation is substantially reduced (Saez et al., 2011). Nuclear entry of PER and TIM requires Importin α1 (IMPα1), which specifically interacts with TIM (Jang et al., 2015). TIM–IMPα1 interaction is abolished by TIM^PL^ (proline 115 to leucine substitution) or TIM^TA^ (threonine 113 to alanine) mutations. Consistently, *tim*^PL^ and *tim*^TA^ flies are arrhythmic and TIM^PL^ remains cytoplasmic in circadian clock neurons (Hara et al., 2011). Additionally, TIM is actively exported from the nucleus by CRM1 and this export is affected by interaction with PER (Ashmore et al., 2003). Another mutation with slower *τ* and abnormal response to light pulses, *tim*^*blind*^, encodes a protein with impaired nuclear accumulation. One of the amino acid substitutions in TIM^blind^ is located within a putative nuclear export signal (NES) (Wülbeck et al., 2005). Collectively, these observations demonstrate the crucial importance of precise regulation of subcellular TIM localization.

Along with light, a primary cue for entrainment, *Drosophila* circadian clocks can be entrained by regular alternations of warmer and colder temperatures (Glaser and Stanewsky, 2005; Sehadova et al., 2009). Also, the distribution of daily activity differs between warm and cold days, which is regulated by temperature-dependent splicing of a *per* intron located within the 3′ untranslated region of mRNA in *D. melanogaster* (Majercak et al., 1999; Zhang et al., 2018). However, at constant conditions, the period length of the circadian clock remains unchanged over a wide range of physiological temperatures. Temperature compensation is a general feature of circadian clocks (Pittendrigh, 1954; Hastings and Sweeney, 1957) conserved from cyanobacteria to mammals (Izumo et al., 2003; Nakajima et al., 2005). In essence, any (bio)chemical reaction runs faster with rising temperature (Arrhenius, 1889), therefore, temperature compensation mechanism should involve multiple reactions, which are differently influenced by temperature, opposing each other (Ruoff, 1992). For example, in the red bread mold, *Neurospora crassa*, temperature-dependent alternative splicing of *frequency* results in long and short FREQUENCY protein isoforms, which have opposing effect on clock speed (Diernfellner et al., 2007). In mammals, distinct phosphorylation of PER2 is important for a temperature-compensated circadian clock (Zhou et al., 2015). Moreover, recently it was shown that the overall activity of the important PER2 kinase CK1δ is temperature-compensated, contributing to temperature-independent τ in the mammals (Shinohara et al., 2017). Interestingly, the *τ* of some period-altering *Drosophila* mutations remains constant over a wide range of temperatures (the circadian clock is well temperature-compensated), whereas others have temperature-dependent phenotypes. In the case of *per*^*L*^, higher temperature further slows down *τ* from 27.8 h at 17°C to 30.5 h at 25°C (Konopka et al., 1989). A similar and even more profound trend was identified in *tim*^*rit*^ where *τ* is 25.5 h at 24°C and rises to 35 h at 30°C (Matsumoto et al., 1999). An opposite temperature compensation abnormality was reported for *per*^*SLIH (Some Like It Hot)*^, a spontaneous mutation frequently found in various laboratory stocks (Hamblen et al., 1998). Interestingly, the *tim*^*SL*^ (*Suppressor of per^*Long*^*) mutation eliminates the temperature compensation defect of *per*^*L*^, whereas *tim*^*SL*^ has no circadian phenotype on its own (Rutila et al., 1996).

Given the length of PER (1218aa) and TIM (1421aa) proteins, however, even the existing remarkable collection of mutants has not been sufficient to uncover all the regions important for the circadian clock machinery and particularly for temperature compensation. Here we discovered that the previously isolated *tim*^*blind*^ allele is defective in temperature compensation similar to the neighboring *tim*^*ritsu*^ allele. To further explore the role of this and other regions of TIM in temperature compensation and clock function, we performed a targeted CRISPR/CAS9 screen, challenging eight different TIM protein regions and isolated ∼20 new mutants with a functional circadian clock, but altered *τ*. Our data revealed that manipulation of one region of TIM in particular, consistently produces temperature compensation defects. In addition, we developed a screening protocol that is an efficient alternative to classical mutagenesis approaches (Price, 2005) or rescue experiments with modified transgenes (Baylies et al., 1992; Landskron et al., 2009).

## Materials and Methods

### Fly Strains and *per*, *cry*, *tim* Combinations

Mutant and wild-type alleles of *per* (*per*^*wt*^, *per*^*S*^, *per*^*T*^, *per*^*SLIH*^, *per*^*L*^) (Konopka and Benzer, 1971; Hamblen et al., 1998), *tim* (*tim*^*wt*^, *tim*^*blind*^, *tim*^*S1*^, *tim*^*L1*^, *tim*^*rit*^, *tim*^*UL*^) (Matsumoto et al., 1999; Rothenfluh et al., 2000a, b, Wülbeck et al., 2005), and *cry* (*cry*^01, 02, 03^, *cry*^*b*^, *cry*^*m*^, and *cry*^*wt*^) (Stanewsky et al., 1998; Busza et al., 2004; Dolezelova et al., 2007) genes were combined by genetics crosses using balancer chromosomes and if necessary, the presence of a particular allele was confirmed by sequencing.

### Locomotor Activity Measurement and Analysis

#### Constant Temperature

Two- to four-days-old males were CO_2_ anesthetized and transferred to 70 mm tubes containing 5% sucrose in agar and loaded into the DAM2 TriKinetics system (Waltham, MA, United States), entrained to light:dark (LD, 12:12) conditions for 5 days and released to constant darkness (DD) for additional 10–14 days. The last 3 days were omitted from the analyses but were used to determine fly survival. To determine *τ* during the first 10 days in DD, chi-square periodogram analysis was performed using ActogramJ (Schmid et al., 2011) and double-plotted actograms were eye inspected in parallel. The same temperature (17, 20, 25, 28°C) was used during the LD entrainment and DD conditions, with the exception of the temperature step-up protocol (see below). For the *tim*^*blind*^, *tim*^*A1128V*^, and *tim*^*L1131M*^ mutations generated by site-directed mutagenesis and homologous recombination, flies were exposed to LD for 3 days, followed by 5–7 days in DD at constant temperatures of 18, 25, or 29°C. Period length and their significance (RS values) were determined using autocorrelation and Chi-square periodogram analysis functions of the fly tool box implemented in MATLAB (MathWorks) (Levine et al., 2002). Period values with associated RS values ≥ 1.5 were considered rhythmic (Levine et al., 2002).

#### Temperature Step-Up Protocol

To be able to measure *τ* in individual flies at different temperatures, 2- to 4-days-old males were CO_2_ anesthetized, loaded into DAM2 TriKinetics system, and entrained in LD (12:12) regime at 20°C for 4 days and then released to DD for 7 days at 20°C (MIR 154 incubators, Panasonic). On eighth day, the temperature was raised to 28°C during 24 h (1°C every 3 h) and locomotor activity was recorded for additional 7 days at 28°C. Step-up protocol was used during the screen to facilitate identification of even subtle temperature-dependent change in *τ* and to enhance throughput during screening.

### Interspecific Comparison of TIM Proteins

Protein sequences of TIM representing dipteran flies *Chymomyza costata* (Kobelkova et al., 2010; Poupardin et al., 2015) and *Musca domestica* (Bazalova and Dolezel, 2017), lepidopteran moth *Ephestia kuehniella* (Kobelkova et al., 2015), heteropteran bug *Pyrrhocoris apterus* used frequently in research of photoperiodic clock (Pivarciova et al., 2016; Urbanova et al., 2016; Kotwica-Rolinska et al., 2017), and basal insect species German cockroach *Blatella germanica* (Bazalova et al., 2016) and firebrat *Thermobia domestica* (Kamae and Tomioka, 2012) were aligned in Geneious 11 using Clustal W algorithm.

### Nuclear Export Signal (NES) Prediction

The putative NESs were identified using motif search in Geneious software according to following consensus patterns after Fung et al. (2015) and Ashmore et al. (2003), respectively, where X corresponds to any amino acid, L = leucine, V = valine, I = isoleucine, F = phenylalanine, M = methionine, and square brackets indicate alternatives:

NES-1a (Fung et al.) [LVIFM]XXX[LVIFM]XX[LVIFM]X [LVIFM]
NES-1b (Fung et al.) [LVIFM]XX[LVIFM]XX[LVIFM]X [LVIFM]
NES-1c (Fung et al.) [LVIFM]XXX[LVIFM]XXX[LVIFM]X [LVIFM]
NES-1d (Fung et al.) [LVIFM]XX[LVIFM]XXX[LVIFM]X [LVIFM]
NES-2 (Fung et al.) [LVIFM]X[LVIFM]XX[LVIFM]X [LVIFM]
NES-3 (Fung et al.) [LVIFM]XX[LVIFM]XXX[LVIFM]XX [LVIFM]
NES-1a-R (Fung et al.) [LVIFM]X[LVIFM]XX[LVIFM]XXX [LVIFM]
NES-1b-R (Fung et al.) [LVIFM]X[LVIFM]XX[LVIFM]XX [LVIFM]
NES-1c-R (Fung et al.) [LVIFM]X[LVIFM]XXX[LVIFM]XXX [LVIFM]
NES-1d-R (Fung et al.) [LVIFM]X[LVIFM]XXX[LVIFM]XX [LVIFM]
  NES (Ashmore et al.) Lx_(1–3)_Lx_(1–3)_Lx[LVIM].

### Prediction of Phosphorylation Sites

The putative phosphorylation sites were predicted *in silico* using NetPhos 3.1 server at http://www.cbs.dtu.dk/services/NetPhos/ and scores higher than 0.5 were plotted in alignments.

### Gene Editing Inducing Non-homologous-End-Joining (NHEJ)—gRNA Design

Target sites were identified using CRISPR target finder^1^ and gRNA design was validated according to parameters mentioned in Ren et al. (2014). To start transcription from U6 promoter 5′ guanine is required; therefore, target sites that lack this feature were extended by single guanine in the 5′ direction (see Table 1 for gRNA sequences and their position on *tim*/TIM). To construct a gRNA expression vector with U6 promoter upstream of *tim*-specific gRNA, two complementary 24-bp oligonucleotides (custom synthesized, Generi Biotech Ltd.) were annealed to obtain a double-strand DNA with 4-bp overhangs compatible to BbsI-linearized pBFv-U6.2 vector (Kondo and Ueda, 2013) obtained from fly stocks of National Institute of Genetics, Japan (NIG-FLY). Plasmid and inserts were ligated with T4 DNA ligase overnight at 4°C and transformed to DH5α competent cells. Presence of the insert was confirmed by PCR and positive clones were sequenced.

**TABLE 1:** List of gRNA used in this study.

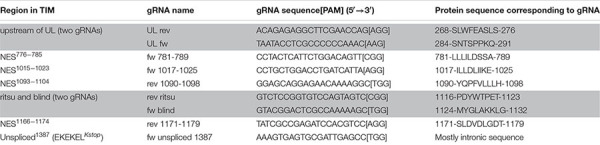

*First column indicates protein region of interest. The name of gRNA specifies its directionality (fw—forward, rev—revers) and position corresponding to protein sequence. Square brackets after each gRNA indicate protospacer adjacent motif (PAM) sequence. Gray background highlights situation when construct contains two gRNAs. Superscript indicates protein sequence resulting from retained intron.*

### Gene Editing Inducing Homology Directed Repair (HDR)—gRNA Design

Target gRNA sites were selected so that Cas9 mediated cleavage was directed to a target locus of 100 bp upstream and downstream of the *tim*^*blind*^ mutation. To avoid off target cleavage optimal target sites were identified using CRISPR target finder (see footnote 1). Two gRNA targets were chosen that are close to the target locus. Complementary target site oligos also contained a 5′ guanine for transcription from the U6 promoter and a 3 bp overhang compatible to BbsI sites. Oligos were annealed using standard primer annealing reactions and cloned into BbsI linearized pCFD3 plasmid (Port et al., 2014) via T4 DNA ligation.

### Gene Editing Inducing HDR—Donor Plasmid Construction

Donor plasmids that contain the desired *tim* point mutations and all elements necessary for homologous recombination were constructed in three subsequent cloning steps. In each round of cloning the 1.5 kb 5′ homology arm and the 1.5 kb 3′ homology arm were individually PCR amplified using outside primers *tim*BMHRF and *tim*BMHRR2 in combination with respective internal primers. Outside primers *tim*BMHRF and *tim*BMHRR2 contain a 15 bp overhang for In-Fusion cloning that is homolog to linearized vector ends. Inside primers have 5′ 15–20 bp extensions that are complementary to each other in addition to one defined mutation for each round of cloning. In the initial round of cloning, Pam site mutations were introduced to avoid unwanted Cas9 cleavage within the donor plasmid. The two fragments (5′ homology arm and 3′ homology arm) were amplified from *y w* flies and assembled into plasmid pBS-KS-attB1-2-PT-SA-SD-0-2xTY1-V5 (Addgene) that was linearized with XbaI and HindIII using In-Fusion cloning. In a second round of cloning, the homology arms were amplified again using the pBS donor plasmid from the previous round as a template. Outside primers were as described above while the inside primers introduced a silent SalI site that can be used to screen for transformants. In-fusion cloning was used to assemble the fragments as described above. The resulting plasmid was then used in a final round of PCR to introduce the individual *tim*^*blind*^ mutations A1128V, L1131M and for remaking the original *tim*^*blind*^ double mutation (see Table 2 for a detailed list of all primers).

**TABLE 2:** Primers used for HDR experiments.

**Primer name**	**Sequence**	**Used for**
timBMHRF	GATGGTCGACTCTAGACGGAGAGTTTGTCAATGACTGC	Outside primer for amplifying 5′ homology region
timBMHRR2	GATGGTCGACAAGCTTCTTGAGACGTAGACGGAGTCGG	Outside primer for amplifying 3′ homology region
TimBMPAMmutF1a	GGAGACAATGTATGGACTCGCCAAAAAGCTGGGAC	Introduces Pam site mutation for gRNA target 1
TimBMPAMmutR1a	AGTCCATACATTGTCTCCGGTGTCCAGTAGT	Introduces Pam site mutation for gRNA target 1
timSalIF	CGTGAGTTAAAGTCGACCACAGAAAAAAACAACCCATTTG	Introduces SalI site
timSalIR	GGTCGACTTTAACTCACGTTTGTCCAGC	Introduces SalI site
timA1128VF	CAATGTATGGACTCGTCAAAAAGCTGGGACCGCT	Introduces A1128V mutation
timA1128VR	GACGAGTCCATACATTGTCTCCGGTGTCCA	Introduces A1128V mutation
timL1131MF	GACTCGTCAAAAAGATGGGACCGCTGGACAAACG	Introduces L1131M mutation for double *tim*^*blind*^ mutant
timL1131MR	CATCTTTTTGACGAGTCCATACATTGTCTCCGG	Introduces L1131M mutation for double *tim*^*blind*^ mutant
timL1131MFa	GACTCGCCAAAAAGATGGGACCGCTGGACAAACG	Introduces L1131M mutation
timL1131MRa	CATCTTTTTGGCGAGTCCATACATTGTCTCCGG	Introduces L1131M mutation
TimBMPAMmutF2a	GACTACTGGACACCAGAAACAATGTATGGACTCGCCA	Introduces Pam site mutation for gRNA target 2
TimBMPAMmutF2b	GACTACTGGACACCAGAAACAATGTATGGACTCGTCA	Introduces Pam site mutation for gRNA target 2
TimBMPAMmutR2a	TTCTGGTGTCCAGTAGTCCGGAATTCTGGCG	Introduces Pam site mutation for gRNA target 2
ScreenTimF1	CTCCCACTTCCGCAACAACAGAGTCTG	Molecular screen of transformants
ScreenTimR2	GCTGCTTACCGAGCTGAGCGAGTTGCG	Molecular screen of transformants
timgRNAT1F	GTCGTGGACACCGGAGACAATGTA	gRNAtarget 1
timgRNAT1R	AAACTACATTGTCTCCGGTGTCCAC	gRNAtarget 1
timgRNAT2F	GTCGCGAGTCCGTACATTGTCTC	gRNAtarget 2
timgRNAT2R	AAACGAGACAATGTACGGACTCGC	gRNAtarget 2

### Transgenesis for NHEJ Mutagenesis

gRNA-encoding plasmids were purified with Qiagen miniprep kit and DNA was eluted in H_2_O. Plasmids were diluted to concentration 100 ng/μl and injected into freshly laid embryos of *y^2^ cho^2^ v^1^ P{nos-phiC31\int.NLS}X; attP2 (III)* stock (NIG-FLY#: TBX-0003) with embryonically expressed phiC31 integrase from transgene located on the X chromosome, attP landing site on the third chromosome, and *chocolate* and *vermillion* (*cho^2^ v^1^*) mutations on the X chromosome. G0 flies were crossed to *y^2^ cho^2^ v^1^; Pr Dr/TM6C, Sb Tb* (NIG-FLY#: TBX-0010) and F1 offspring were selected for eye color rescue (*v*^+^ transgene in the *cho^2^ v^1^* background turns the eye color from light orange to dark brown). Strains with gRNA-encoding transgene were balanced with TM6C, *Sb Tb* and kept as stock.

### Transgenesis for HDR Experiments

Donor plasmids containing the desired mutation along with gRNA plasmids were verified by sequence analysis and scaled up for injections using Qiagen plasmid midiprep; 6 μg of each plasmid was precipitated and eluted in injection buffer. gRNA construct and donor plasmids were mixed prior to injection and the mix was injected into freshly laid embryos of *nos*-Cas9 flies (Port et al., 2014). Surviving adults were backcrossed in batch crosses to *y w*, *Bl/CyO*, + flies to balance second chromosome modifications with CyO. Individual male and female flies from this cross were crossed again to *y w*, *Bl/CyO*, +. After egg deposition for 3–5 days, adult transformant flies were used for molecular screening.

Because initial attempts to introduce the A1128V mutation did not result in any positive transformants, a slightly different approach was used. The original *tim*^*blind*^ EMS stock was crossed to nosCas9 flies and embryos of this cross were injected with donor plasmids containing the A1128V mutation and the wild-type residue at position 1131 to back mutate the M at this position to L.

### Genetic Crosses Inducing NHEJ

The CAS9 editing procedure utilized fly strains and tools established by Kondo and Ueda (2013). Flies expressing Cas9 specifically in germ cells (nos-Cas9) from third chromosome insertion (NIG-FLY#: CAS-0003; *y^2^ cho^2^ v^1^*; *P{nos-Cas9*, *y+*, *v+}3A/TM6C*, *Sb Tb*) were crossed with individual U6gRNA-encoded transgenic strains (also located on the third chromosome). Resulting offspring thus expressed both gRNA and CAS9 on third chromosome, which potentially targeted *tim* gene located on the second chromosome and induce insertions and deletions as a result of non-homologous-end-joining (NHEJ) mechanism. The resulting offspring were crossed to *y^2^ cho^2^ v^1^*; *Sco/CyO* (NIG-FLY#: TBX-0007) to balance modified second chromosome by *CyO*. Males and females with second chromosome balancer were individually crossed again to *y^2^ cho^2^ v^1^*; *Sco/CyO* flies to establish lines with identically modified second chromosomes (see Supplementary Figure S1 for the crossing scheme).

### Behavioral Screening in NHEJ Experiments

To identify mutants, locomotor activity of eight males per line (homozygous for the second chromosome) was recorded in temperature step-up protocol and any alternations in locomotor activity pattern (arrhythmicity, change in *τ*) were identified from the double-plotted actograms. In parallel, reference strains with either functional (*w*^1118^ or Canton S) or altered temperature compensation (*tim*^*rit*^) were recorded as a negative and positive control. Phenotypes of putative mutant lines were further confirmed on a large sample in temperature step-up protocol and also independently at low (17°C), ambient (25°C), and high (28°C) constant temperatures.

### Molecular Screening in HDR Experiments

A total of 95 flies for each mutation were screened using PCR and restriction digests. In detail, a ∼800 bp target locus containing the expected mutations along with the introduced SalI restriction site was amplified by PCR using genomic DNA from individual flies; 1 μl of SalI was then added to half of the PCR and incubated for 2 h at 37°C. Resulting products were analyzed on agarose gels. The remaining PCR product of samples that showed digested products of the correct size was then used for sequencing to verify the presence of the desired mutations.

### Sequencing of Mutated Region

To characterize the molecular nature of the CRISPR/Cas9-induced mutations, target loci were first amplified by PCR using genomic DNA extracted from individual flies. One fly was crushed in 50 μl of Squishing buffer (10 mM Tris-HCl pH8; 1 mM EDTA; 25 mM NaCl; 200 g/ml Proteinase K), followed by incubation at 37°C for 30 min and later Proteinase K inactivation at 95°C for 3 min. Using 1 μl of the crude DNA extract as a template, a DNA sequence surrounding the target site was amplified by PCR for 35 cycles in a 10 μl reaction with 2X PPP Master Mix (Top-Bio, Prague, Czech Republic). The PCR products were analyzed by agarose gel electrophoresis and Sanger sequencing. Primers used for PCR and DNA sequencing are listed in Table 3 and the actual number of obtained mutants is in Table 4.

**TABLE 3:** Primers used for amplification and subsequent sequencing of mutants.

**Targeted region**	**Note**	**Orientation**	**Primer sequence**	**Amplicon size (bp)**
Upstream of UL	Used for sequencing	Forward	ACTCCTGTATCTGATGACC	334
		Reverse	GATACTCCTGACCCTTGC	
	Used for detection of the in/del only	Forward	TACAAGGATCAGCATGTG	185
		Reverse	GCCATTGCTGCCATTGT	
NES^776–785^		Forward	GCGAAATGTCCGATCTGAGG	188
		Reverse	CCCTACTGTGTTATGTGCTC	
NES^1015–1023^		Forward	CCTCAGATGATGTTCAGGTG	156
		Reverse	GCAGCACTCAATGAGGATCC	
NES^1093–1104^		Forward	CCGGAAGGCGATCACATCAT	217
		Reverse	GTCCGTACATTGTCTCCGGT	
ritsu and blind	Used for detection of the in/del only	Forward	GCAGTGGAACAACGAGCAAT	225
		Reverse	TGTGGAATGACAAATGGGT	
	Used for detection of the in/del only	forward	CTCCACAAGCTGGGCATT	132
		Reverse	CTTTAACTCACGTTTGTCCAGC	
	Used for sequencing	Forward	GATCACATCATGGAGCCGGTG	565
		Reverse	TGAGCGAGTTGCGGGGTC	
NES^1166–1174^		Forward	CCTCAAGTTCGACGCCAGTG	238
		Reverse	GTTGCAGTGCTTCGTCTTGG	
Unspliced^1387^		Forward	GGCTGGAAATGGATGTGGAC	280
		Reverse	CTGTCAAACTGAGAGGTGAC	

**TABLE 4:** Summary of screened lines and identified mutants.

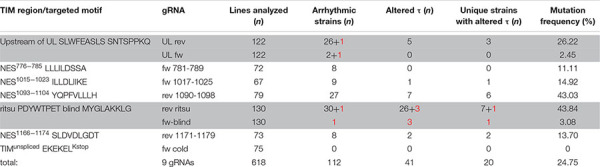

*Gray background refers to situation when two gRNAs were used simultaneously (double gRNA construct). Red color highlights mutants resulting from cleavage directed by both gRNAs. Mutation frequency refers to mutants with altered circadian phenotypes, whereas actual frequency of mutations at DNA level was not determined. Superscript indicates protein sequence resulting from retained intron.*

### Immunocytochemistry

Seven-days-old males kept in LD regime and constant temperature of 17, 20, or 28°C were collected at specific time points (every 4 h) during the day; red light was used for collection in the dark time points. Flies were fixed in 4% paraformaldehyde in phosphate buffer saline (PBS; pH 7.4) for 1.5 h, washed in PBS twice, and brains were dissected. Next, tissues were fixed again for 45 min in 4% PFA and washed five times in PBS with an addition of 0.2% Triton X-100 (PBT). After that, brains were incubated in 5% normal goat serum (NGS) with an addition of 0.5% bovine serum albumin (BSA) for 30 min first at room temperature, and then incubated with primary anti-PER antibodies (rabbit, 1:5000, Stanewsky et al., 1997) for 3 days and with anti-PDF (mouse, 1:500, Hybridoma Bank) for 1 day at 4°C. Afterward, brains were washed six times in PBT/BSA and blocked in 5% NGS for 45 min. After that, goat anti-rabbit (conjugated with Cy3, 1:500, Jackson Immuno Research) and goat anti-mouse (conjugated with Alexa 488, 1:1000, Molecular Probes) secondary antibodies were applied overnight in 4°C. Finally, brains were washed twice in BSA, six times in PBT, and twice in PBS. Then, brains were mounted in Vectashield medium (Vector) and examined under a Zeiss Meta 510 laser scanning microscope. The identical laser settings were used for all images.

### Quantitative Comparison of Immunofluorescence Values

Collected images were analyzed using ImageJ software (NIH, Bethesda). PDF-immune-positive cells were marked (green channel) and PER fluorescence intensity (red channel) was measured in the selected area and outside of the PDF-expressing cells (background). Fluorescence intensity is represented by the mean gray value (the sum of the values of all pixels in the area divided by the number of pixels within the selection). The final value was obtained by subtracting the background value from the staining in the selected area. At least 10 brains per each time point were checked. Experiments were repeated three times.

### Compensation for Different Staining Affinity After Antibody Re-use

Brains from every genotype were isolated at ZT0 at 17, 25, and 28°C in parallel. After immunostaining, mean gray value was measured. The mean was used to calculate the ratio between the intensity staining in different temperatures for every genotype separately. Then the ratio between means at ZT0 at different temperatures from previous experiments was calculated, which allowed identifying the factor used for data compensation. This allowed us to avoid differences in staining intensity caused by changes in antibodies affinity after re-use.

### Statistical Analysis

The differences between *τ* were tested for statistical significance by one-way ANOVA with Tukey–Kramer’s *post hoc* test using Graphpad7 (Prism) software. ICC staining intensities were compared by one-way ANOVA between genotypes for each timepoint, temperature, and cell type.

## Results

### Genetic Interaction Between *tim*, *cry*, and *per*

We combined existing *cry* alleles (*cry*^01, 02, 03^, *cry*^*b*^, *cry*^*m*^, and *cry*^*wt*^) with available alleles of *per* (*per*^*wt*^, *per*^*S*^, *per*^*T*^, *per*^*SLIH*^, *per*^*L*^) and *tim* (*tim*^*wt*^, *tim*^*blind*^, *tim*^*S1*^, *tim*^*L1*^, *tim*^*rit*^, *tim*^*UL*^). Locomotor activity was recorded in 65 genetic combinations at low (18°C), standard (25°C), and high (28°C) temperatures. In general, the observed free running periods were consistent with published data including previously reported temperature compensation defects of *per*^*L*^ (Konopka et al., 1989), *tim*^*rit*^ (Matsumoto et al., 1999), and *per*^*SLIH*^ (Hamblen et al., 1998). Similarly, combination of *per*^*L*^ with *cry* mutants (*cry*^01, 02, 03^, *cry*^*b*^, *cry*^*m*^) resulted in flies with temperature compensation defects comparable to *per*^*L*^ alone (Fexová, 2010, M.Sc. thesis, and Supplementary Table S1), in agreement with the retraction note for Kaushik et al. (2007) (PLoS Biol 2016, 14:e1002403).

### *tim*^*blind*^ Is a Temperature Compensation Mutant

**FIGURE 1:**
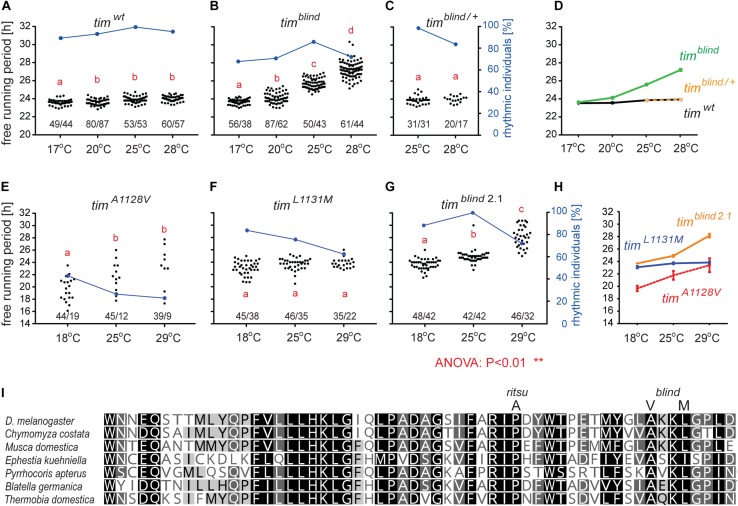
*timeless*^blind^ is a circadian clock mutant with defect in temperature compensation [each dot in panels **A–C** and **E–G** represents the free running period (*tau*, *τ*) of individual male flies recorded during 10 days in DD at the specified temperature]. Blue dots connected with a line indicate the percentage of rhythmic individuals (scale is on the right *y*-axis) at a particular temperature). **(A)** *τ* of control Canton S wild-type (wt) flies, **(B)** *tim*^*blind*^ homozygous, and **(C)** *tim^*blind/*^^+^* heterozygous *D. melanogaster* males in constant darkness (DD) and constant temperature (17, 20, 25, and 28°C). **(D)** Comparison of *τ* (shown as mean ± SEM) for all three genotypes indicates the temperature-dependent *τ* lengthening in *tim*^*blind*^ and confirms that the mutant is fully recessive. **(E)** *tim*^A1128V^, a mutant with only the first *tim*^*blind*^ position mutated, is mostly arrhythmic with a potential temperature compensation defect in rhythmic individuals, **(F)** *tim*^L1131M^, a mutant with only the second *tim*^*blind*^ position mutated, exhibits normal and fully temperature-compensated τ. **(G)** The synthetic *tim*^*blind2.1*^ allele shows temperature compensation defects and solid rhythmicity. **(H)** Comparison of *τ* (shown as mean ± SEM) for re-engineered single and double-mutant versions of *tim*^*blind*^. **(I)** Alignment of various insect TIM proteins near TIM^rit^ and TIM^blind^ mutations (specific substitution in *rit* and *blind* is shown above the alignment). Insects are ordered according to their phylogenetic relationship from the most diverged at the top to the most ancestral at the bottom, with the following insect groups: Diptera (*Drosophila*, *Chymomyza* and *Musca*), Lepidoptera (*Ephestia*), Heteroptera (*Pyrrhocoris*), Blattodea (*Blatella*), and Ametabola (*Thermobia*). A, V, and M refer to the single letter aminoacid code.

*tim*^*blind*^ was identified in a chemical mutagenesis screen for mutations altering *period* gene expression and contains two conservative amino acid substitutions, alanine to valine (V) at position 1128 and leucine (L) to methionine (M) at position 1131 (Wülbeck et al., 2005). While *tim*^*blind*^ flies exhibit a long (26 h) free running period length of locomotor activity (measured at 25°C), the period length of eclosion rhythms (measured at 20°C) is normal (24.5 h) (Wülbeck et al., 2005). We therefore tested the possibility that *tim*^*blind*^ mutants are defective in temperature compensation. Indeed, *tim*^*blind*^ showed a normal *τ* of locomotor activity rhythms at 17°C (23.7 h), and gradually longer *τ* at higher temperatures (24.0 h at 20°C; 25.7 h at 25°C; 26.8 at 28°C; *P* < 0.001, Figures 1B,D and Table 5), confirming that this *tim* allele affects temperature compensation. In agreement with Wülbeck et al. (2005), the mutant is fully recessive (Figures 1C,D). One of the two *tim*^*blind*^ amino-acid substitutions (L1131M) maps to one of the six potential NESs originally predicted for TIM (Ashmore et al., 2003), indicating that this mutation is responsible for the *tim*^*blind*^ phenotypes (Wülbeck et al., 2005). To test this hypothesis, we introduced the individual substitutions as well as the double-mutant into the endogenous *tim* gene, using site-directed mutagenesis combined with CRISPR/Cas9 mediated homologous recombination (Port et al., 2014; see section “Materials and Methods”). Locomotor behavior of the resulting mutants (*tim*^*A1128V*^, *tim*^*L1131M*^, *tim*^*blind–2.1*^) was analyzed in DD at 18, 25, and 29°C to determine rhythmicity and potential defects in temperature compensation. Surprisingly, none of the single mutants recapitulated the phenotypes of the original *tim*^*blind*^ double-mutant. The *tim*^*A1128V*^ mutation led to high percentage of arrhythmicity, ranging from 57% at 18°C to 77% at 29°C (Figure 1E and Table 5). Although the remaining rhythmic flies had variable periods at all three temperatures, overall they still indicate a potential period lengthening with increasing temperatures (Figures 1E,H and Table 5). The severe impact of the *tim*^*A1128V*^ mutation on rhythmicity is surprising, given the conservative nature of this amino acid replacement. In contrast, the predicted NES mutation *tim*^*L1131M*^ basically had no effect on rhythmicity and period length at any temperature. Between 84% (18°C) and 63% (29°C) of the mutant flies were rhythmic with period values slightly below 24 h at all temperatures (Figures 1F,H and Table 5). On the other hand, the re-engineered *tim*^*blind*^ double-mutant (*tim*^*blind–2.1*^) showed temperature-dependent period lengthening and robust rhythmicity at all temperatures, closely matching the phenotypes of the *tim*^*blind*^ EMS allele (Figures 1G,H and Table 5, Wülbeck et al., 2005). At 25°C, the period length of *tim*^*blind–2.1*^ is about 1 h shorter compared to *tim*^*blind*^ (Figures 1B,G and Table 5, Wülbeck et al., 2005), while at 29°C *tim*^*blind–2.1*^ it is about 1 h longer compared to *tim*^*blind*^ at 28°C. Because the experiments with the original *tim*^*blind*^ allele and *tim*^*blind–2.1*^ were performed in different laboratories (České Budějovice and Münster, respectively), small differences in the experimental set-up (e.g., temperature), or period length determinations could explain these discrepancies. Importantly, both the synthetic and original *tim*^*blind*^ alleles show a pronounced defect in temperature compensation, and we show here that both amino acid substitutions are required to elicit this phenotype.

**TABLE 5:** Summary of circadian phenotypes in new mutants and reference lines.

		**17°C**				**20°C**				**25°C**				**28°C**			
					
**TIM region**	**Genotype**	**(*n*)**	**Rhythm %**	**FRP (h)**	**SEM**	**(*n*)**	**Rhythm %**	**FRP (h)**	**SEM**	**(*n*)**	**Rhythm %**	**FRP (h)**	**SEM**	**(*n*)**	**Rhythm %**	**FRP (h)**	**SEM**
	wt-CS (Canton S)	56	89.80	23.59	0.05	88	93.75	23.62	0.04	66	100.00	23.91	0.05	72	95.00	23.99	0.04
	CS/*tim*^01^	30	96.67	32.66	0.06	26	96.15	23.78	0.05	25	100.00	23.95	0.06	28	92.86	23.57	0.05
Upstream of UL	*tim*^ΔW270^	48	80.43	25.25	0.05	56	93.75	25.41	0.06	48	92.50	25.74	0.10	48	86.67	25.55	0.07
	*tim*^ΔW270^/+	32	93.55	24.62	0.07	32	100.00	24.22	0.05	32	100.00	24.45	0.04	32	76.67	24.74	0.08
	*tim*^ΔW270^/*tim*^01^	32	100.00	25.31	0.05	32	100.00	25.69	0.06	32	96.43	25.86	0.07	32	96.67	26.32	0.09
	*tim*^W270Y^	32	90.32	23.04	0.06	48	97.87	22.97	0.05	40	92.50	22.95	0.05	47	97.87	23.13	0.05
	*tim*^ΔW270Y^/+	32	93.75	22.89	0.06	32	93.33	22.73	0.05	32	100.00	23.34	0.06	32	79.31	22.93	0.08
	*tim*^ΔW270Y^/*tim*^01^	32	100.00	22.91	0.04	32	96.15	23.21	0.05	32	96.77	23.30	0.08	64	96.36	23.33	0.07
	*tim*^W270YYY^	56	21.82	24.48	0.14	48	53.49	24.81	0.07	56	19.61	25.14	0.28	48	11.90	26.95	0.43
	*tim*^ΔW270YYY^/+	32	93.10	24.19	0.08	32	100.00	24.03	0.05	32	96.67	24.03	0.09	32	93.33	24.52	0.16
	*tim*^ΔW270YYY^/*tim*^01^	32	93.33	24.55	0.05	32	100.00	24.73	0.06	32	100.00	25.61	0.07	32	82.76	26.61	0.17
NES^1017–1025^	*tim* ^ΔI1022^	48	87.23	23.04	0.06	48	97.73	23.34	0.04	16	100.00	23.23	0.05	32	80.65	23.27	0.10
	*tim* ^ΔI1022^/+	32	96.55	23.39	0.06	64	95.16	23.42	0.03	48	93.62	23.29	0.07	32	93.55	23.01	0.08
	*tim* ^ΔI1022^/*tim*^01^	32	93.75	23.74	0.04	32	93.75	23.70	0.04	32	100.00	23.42	0.07	32	93.33	23.11	0.07
NES^1093–1104^	*tim*^ΔLYQPFVLLLHK1089–99^	40	47.37	24.56	0.07	48	43.48	24.77	0.18	48	81.82	28.09	0.14	48	12.77	30.75	0.71
	*tim*^ΔLYQPFVLLLHK1089–99^/+	48	90.48	23.27	0.05	32	96.67	23.54	0.06	48	93.75	24.01	0.06	32	90.00	23.88	0.05
	*tim*^ΔLYQPFVLLLHK1089–99^/*tim*^01^	32	84.38	23.82	0.10	32	82.76	24.19	0.10	40	94.87	26.38	0.17	32	31.25	30.61	0.38
	*tim*^ΔPFVLL1092–96^	56	50.94	25.13	0.10	48	64.58	24.61	0.08	56	86.79	26.29	0.06	48	45.83	30.72	0.16
	*tim*^ΔPFVLL1092–96^/+	32	84.38	23.45	0.05	32	93.33	23.31	0.05	32	96.88	23.66	0.04	32	100.00	24.74	0.14
	*tim*^ΔPFVLL1092–96^/*tim*^01^	49	89.58	23.71	0.07	32	96.43	23.82	0.08	48	97.62	25.48	0.08	36	72.73	28.92	0.29
	*tim*^ΔPFVLLL1092–97;K1099L^	80	72.15	24.74	0.05	48	76.60	25.08	0.06	66	83.93	28.37	0.08	80	0.00		
	*tim*^ΔPFVLLL1092–97;K1099L^/+	32	81.25	23.64	0.02	32	81.48	24.10	0.09	48	95.24	24.59	0.06	32	92.59	24.56	0.06
	*tim*^ΔPFVLLL1092–97;K1099L^/*tim*^01^	32	89.29	24.08	0.13	32	78.13	24.68	0.14	64	76.67	27.10	0.16	32	0.00		
	*tim*^ΔFV1093–94^	32	81.25	23.61	0.08	48	95.74	24.06	0.05	48	82.93	27.66	0.13	48	12.77	31.54	0.05
	*tim*^ΔFV1093–94^/+	32	87.50	23.22	0.05	32	100.00	23.49	0.05	32	96.67	23.82	0.07	32	96.67	24.86	0.22
	*tim*^ΔFV1093–94^/*tim*^01^	32	77.42	23.62	0.09	32	96.67	23.89	0.06	32	87.50	25.96	0.13	40	55.88	27.35	0.67
ritsu	*tim*^rit^	48	89.74	24.82	0.28	80	97.67	25.06	0.31	32	93.33	26.08	0.33	64	84.75	28.86	0.82
	*tim^*rit*^/+*	28	100.00	24.11	0.06	15	100.00	24.03	0.03	16	100.00	24.28	0.06	14	100.00	24.35	0.03
	*tim^*rit*^/tim*^01^	28	100.00	23.53	0.12	29	100.00	24.41	0.04	30	100.00	25.51	0.05	32	100.00	27.57	0.18
	*tim*^ΔFARIPD1112–17^	48	14.58	24.55	0.22	48	42.55	25.13	0.08	48	52.08	30.16	0.14	56	0.00		
	*tim*^ΔFARIPD1112–17^/+	32	48.39	23.42	0.09	32	90.32	23.57	0.06	32	100.00	23.62	0.06	32	100.00	24.41	0.09
	*tim*^ΔFARIPD1112–17^/*tim*^01^	32	65.52	22.89	0.12	32	86.21	24.06	0.09	48	87.50	27.04	0.21	32	0.00		
	*tim*^ΔPDYWT1116–20^	48	71.74	24.44	0.08	48	70.21	24.73	0.10	32	79.31	27.42	0.17	80	16.00	30.28	0.36
	*tim*^ΔPDYWT1116–20^/+	62	93.33	23.42	0.06	32	96.88	23.38	0.06	48	100.00	23.93	0.04	36	97.06	24.55	0.08
	*tim*^ΔPDYWT1116–20^/*tim*^01^	32	87.50	23.30	0.08	32	96.88	24.05	0.07	32	80.65	26.23	0.23	40	66.67	30.09	0.23
	*tim* ^ΔY1118^	49	60.87	23.95	0.08	48	93.48	23.99	0.04	40	94.44	24.65	0.05	48	71.11	25.55	0.08
	*tim* ^ΔY1118^/+	32	90.63	23.51	0.06	32	100.00	23.61	0.06	32	90.32	24.10	0.09	32	96.30	24.29	0.08
	*tim* ^Δ*Y1118/*^*tim*^01^	32	96.77	23.61	0.05	32	100.00	24.12	0.04	32	100.00	24.48	0.08	48	100.00	25.20	0.10
NES^1166–1174^	*tim*^ΔLD1172–73^	48	50.00	23.43	0.04	48	65.22	23.67	0.05	16	40.00	23.57	0.10	48	50.00	23.18	0.06
	*tim*^ΔLD1172–73^/+	32	83.33	23.46	0.07	32	100.00	23.50	0.06	32	100.00	23.29	0.07	32	91.67	23.14	0.12
	*tim*^ΔLD1172–73^/*tim*^01^	40	93.94	23.80	0.05	32	100.00	23.67	0.06	40	90.63	23.93	0.08	32	93.75	23.16	0.07
	*tim*^ΔDVDLG1173–77^	40	71.79	23.24	0.06	56	80.77	23.67	0.05	10	80.00	23.60	0.10	32	48.15	24.26	0.18
	*tim*^ΔDVDLG1173–77^/+	32	90.32	23.62	0.05	32	100.00	23.68	0.04	32	96.77	23.93	0.05	32	100	23.58	0.07
	*tim*^ΔDVDLG1173–77^/*tim*^01^	32	96.77	23.58	0.05	32	100.00	24.33	0.05	32	96.88	24.20	0.06	64	94.643	24.75	0.11
blind	*tim*^1118AR;Δ12aa^	112	64.52	24.95	0.09	48	70.73	25.24	0.18	64	68.33	27.92	0.15	88	3.70	29.42	1.00
	*tim*^1118AR;Δ12aa^/+	32	66.67	23.68	0.10	32	83.87	23.81	0.09	80	100.00	24.58	0.04	48	97.83	24.72	0.08
	*tim*^1118AR;Δ12aa^/*tim*^01^	32	73.08	24.21	0.15	32	73.68	25.20	0.15	40	66.67	28.10	0.20	32	0.00		
	*tim*^blind^	83	72.29	23.71	0.05	104	70.77	24.04	0.08	56	86.00	25.65	0.08	80	72.13	26.80	0.27
	*tim*^blind^/+	30	100.00	23.85	0.09	11	100.00	23.63	0.09	32	100.00	23.89	0.08	23	85.00	23.99	0.09
	*tim*^blind^/*tim*^01^	30	83.33	23.18	0.08	30	100.00	23.93	0.03	30	100.00	25.00	0.05	30	96.67	26.19	0.13
		18°C								25°C				29°C			
blind	*tim*^A1128V^	44	43.18	21.67	1.02	–	–	–	–	45	26.67	22.54	0.97	39	23.07	24.70	0.92
	*tim*^blind^ ^2.^*^1^*	48	87.50	23.74	0.91	–	–	–	–	42	100.00	24.88	0.13	46	69.57	27.61	0.16
	*tim*^L1131M^	45	84.44	23.22	0.19	–	–	–	–	46	76.09	23.70	0.16	35	62.86	23.82	0.17

*Rhythm % indicates percentage of rhythmic males.*

Interestingly, in both *tim* temperature compensation alleles, *tim*^*rit*^ and *tim*^*blind*^, the mutations are located in the same region of the TIM protein, separated by only 11–14 amino acids (Figure 1I). TIM^rit^ was isolated from a natural fruitfly population and harbors a proline (P) to alanine (A) amino acid substitution at position 1093 (Matsumoto et al., 1999) which corresponds to position 1116 in the L-TIM protein, where additional 23 amino acids are added at the N-terminus (Tauber et al., 2007). Protein alignment indicates that these TIM regions are highly conserved across even distantly related insect species, including hemimetabolan *P. apterus*, *B. germanica*, and ametabolan *T. domestica* (Figure 1I).

The most common NES motif that is recognized by CRM1 is best described by the consensus sequence L-X(2,3)-[LIVFM]-X(2,3)-L-X-[LI], where X(2,3) represents any two or three amino acids that separate the four key hydrophobic residues. Although a large amount of proteins contain this consensus sequence, only a small percentage of NESs are actually functional. On the other hand, only 36% of experimentally identified functional NESs match this consensus (Kosugi et al., 2008). Thus, predicting functional NES motives is still challenging and a reliable approach has not yet been described. Therefore, we used different approaches to analyze potential NES motives in TIM.

**FIGURE 2:**
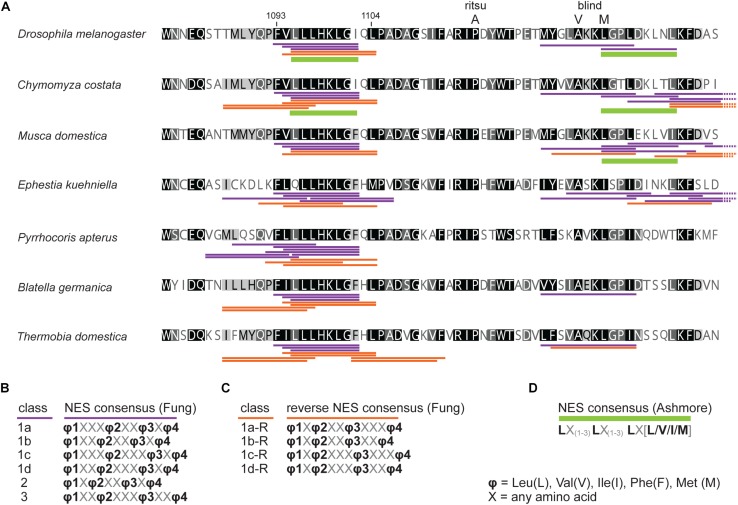
*In silico* prediction of nuclear export signals (NESs) in TIM^rit^ vicinity. **(A)** Protein alignment (ancestral species are at the bottom) with underlined NES motifs. Purple and orange colors code for forward and reverse NES classes, respectively, and green bar indicates strict NES prediction after Ashmore et al. (2003). **(B–D)** The consensus sequences of NES classes. Numbers above the alignment correspond to amino acid residues in L-TIM (*D. melanogaster*).

First, we assess the evolutionary conservation of NES in TIM by identifying and comparing NES motifs in the above-mentioned “primitive” insect species and included two additional dipteran (*C. costata* and *M. domestica*) and one lepidopteran (*E. kuehniella*) representatives. We searched for strict NES motifs using the consensus after Ashmore et al. (2003). Six NES motifs were found in the entire *Drosophila* TIM, and two of them located near the TIM^blind^ and TIM^rit^ mutations. The NES 1031-1139, which overlaps with TIM^blind^, is further conserved in all Diptera (Figure 2). The second NES, located upstream of TIM^rit^ (residues 1095-1012), is apart from *D. melanogaster* only found in the drosophilid fly *C. costata*. To identify additional putative NES in the TIM^blind^ and TIM^rit^ regions, we applied less strict motifs as described in Fung et al. (2015). Multiple NES motifs were found in *D. melanogaster* near the TIM^blind^ and TIM^rit^ mutations, all of them at least partially overlapping with the two strict “Ashmore’s” NES (Figure 2A). Using the less rigid “Fung” consensus, NES motifs were even found in species for which the strict consensus did not reveal any NES. Importantly, these less strict NES motifs are still present in homologous sequences (Figure 2).

We hypothesized that the fact that both *tim* temperature compensation mutants, *tim*^*rit*^ and *tim*^*blind*^, are spaced close to each other is not merely accidental, as all the other known *tim* mutations that are dispersed throughout the *tim* gene region have not been reported to have a temperature-dependent phenotype. To test this assumption, we decided to conduct a targeted mutagenesis screen for temperature compensation mutations in *tim*.

### Step-Up Protocol for Detection of Temperature Compensation Mutants

**FIGURE 3:**
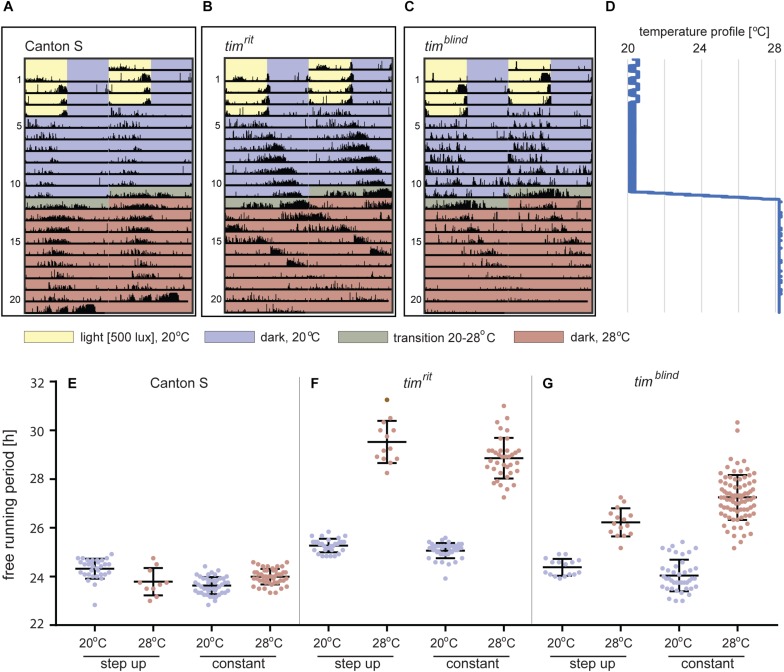
Locomotor activity of *D. melanogaster* at different temperatures. **(A–C)** Typical double-plotted actograms obtained during the step-up protocol consisting of 4-day LD entrainment at 20°C followed by 7 days of DD at 20°C. Then, temperature was raised to 28°C during 16 h (1°C every 2 h) and kept constant for the rest of the experiment. **(D)** Temperature profile recorded during the step-up protocol (note small thermoperiodic oscillations typical for LD conditions). **(E–G)** Comparison of the free running periods (*τ*) measured at low and high portions of the step-up protocol, respectively, with data obtained from flies entrained and recorded at one constant temperature. Each dot corresponds to FRP of individual male fly, and colors correspond to the temperature-coding used in actogram backgrounds. Not all males survived entire step-up experiment (note lower *n* at 28°C). Bars show the mean ± SD.

To unambiguously determine and compare *τ* at different temperatures in individual flies, we developed and optimized a protocol for assaying locomotor activity in flies exposed to two different temperatures. Fruit flies can survive for more than 2 weeks in the glass tubes (5 mm diameter, 70 mm length, and at least 1 cm of agar with 5% sucrose) used in DAM2 monitors, so we decided to record their activity at low temperature for 7 days followed by further 7 days at high temperature providing enough data for each condition in a single fly (Figures 3A,B,D). The transition from the low to the high temperatures was experimentally optimized to an 8°C increase spread over 24 h (this gradual temperature rise was programmed in MIR 154, Panasonic, as eight successive steps, each 2 h long with 1°C increase) (Figures 3A,B,D). We verified that the *τ* values measured in this step-up protocol are comparable to values obtained at constant temperatures (Figures 3E–G). Representative actograms in Figure 3C illustrate that even the relatively subtle *τ* change of *tim*^*blind*^ can be easily spotted by eye and so that this approach is suitable for efficient screening of large datasets for altered circadian phenotypes.

### CRISPR/CAS9 Targeted Mutagenesis of *tim*

**FIGURE 4:**
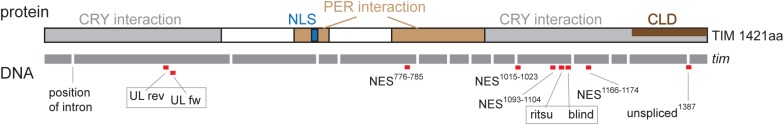
Schematic depiction of TIM protein with highlighted functional domains and interacting regions: nuclear localization signal (NLS), PERIOD interacting domain (PER interaction), CRYPTOCHROME interacting domain (CRY interaction), and cytoplasmic localization domain (CLD). Below, schematic depiction of DNA is shown with exons in gray (length of the bar corresponds to exon length) separated by introns (the length is not shown). Red boxes indicate position of gRNA; the number in gRNA corresponds to amino acid residues in the TIM protein. Gray frame indicates when two gRNAs were used simultaneously in one construct (UL rev + UL fw; ritsu + blind).

Seven regions of TIM were selected for targeted mutagenesis based on phylogenetic conservation analyses and/or their position with respect to known temperature compensation mutations (Figure 4): (a) a conserved sequence motif located in the first quarter of the protein near the UL mutation, (b) TIM^unspliced^ comprising the last intron which is alternatively retained at low temperatures (Boothroyd et al., 2007; Montelli et al., 2015), (c) the area where TIM^rit^ and TIM^blind^ mutations are located, (d) NES^1093–1104^, a conserved region 12-23 aa upstream of TIM^rit^, (e) NES^1015–1023^ motif 93-98 aa upstream of TIM^rit^, (f) NES^1166–1174^ motif 56-62 aa downstream of TIM^rit^, and (g) NES^776–785^ located approximately in the middle of TIM (see Table 1 for gRNA sequences, cleavage sites and corresponding sequences and Figure 4 for position of selected regions on TIM).

Corresponding gRNA expressing plasmids were cloned, verified, and stably transformed into the attP2 landing site on the third chromosome following an established protocol (Kondo and Ueda, 2013). The targeted cleavage of genomic DNA within the *tim* gene region was induced in the embryonic stage by combining the nosCAS9 and U6gRNA-expressing transgenes to create in/dels resulting from NHEJ mechanism. The targeted chromosomes were balanced and subsequently brought into homozygosity (see Supplementary Figure S1 for genotypes and genetic crosses and Supplementary Figure S2 for *τ* of used *Drosophila* lines and lines suitable for similar experiments). In total, 618 lines covering seven *tim* regions were established and screened for altered circadian rhythmicity using our step-up protocol (Table 4).

Both arrhythmic mutants and mutant lines displaying altered rhythmicity were identified in the screen. In total, we isolated 113 arrhythmic lines of which a subset was molecularly characterized. In all cases, this uncovered out-of-frame in/del mutations resulting in premature stop codons. In contrast, *τ*-altering mutations always contained in-frame modifications. Deletions were approximately 15 times more frequent than insertions, the length of deletions ranged from 1 to 71 bp, insertions were 2 to 8 bp long, and one-third of in/dels were combined with a substitution (see Supplementary Figure S3 for DNA sequences of isolated mutants); 35 behaviorally normal flies were also sequenced and no modifications were observed.

## Functional Characterization of Novel Mutant Lines

The step-up protocol was efficient for quick identification of putative mutants with either arrhythmic behavior or with a change in *τ* including even small temperature-dependent lengthening. However, for precise phenotype assessment, follow-up experimental replicates were performed allowing us to determine the accurate percentage of rhythmicity associated with each mutation, the exact *τ* at four tested temperatures, and the phenotypes in heterozygous conditions (heteroallelic combinations with wild-type *tim* and *tim*^01^).

### Region Upstream of TIM^UL^

**FIGURE 5:**
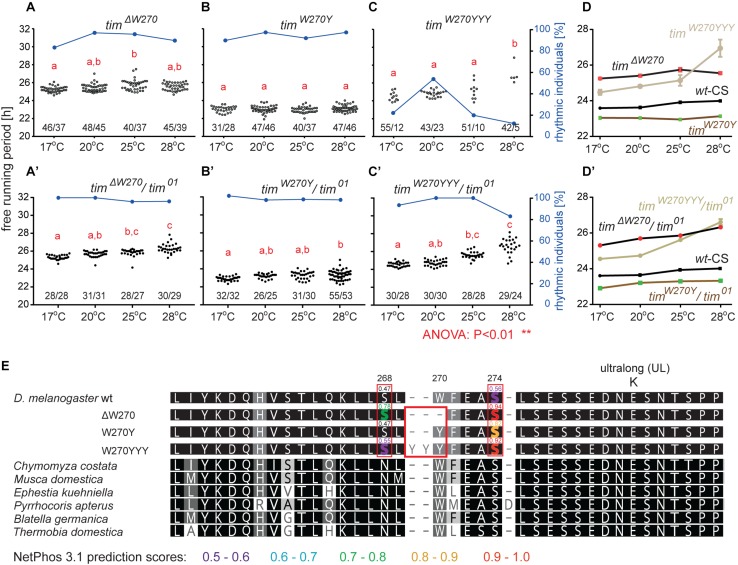
Mutants isolated upstream of *tim*^*UL*^. **(A–C)** Each dot represents *τ* of individual fly as was recorded during 10 days in DD and specified temperature. Lowercase red letters above dots indicate a significant difference between “a” and “b” and “c” and “d” (one-way ANOVA with Tukey–Kramer’s *post hoc* test, *P* < 0.01). Numbers below dots correspond to number of measured/rhythmic flies at particular temperature. Blue dots connected with line correspond to percentage of rhythmic individuals (scale on right *y*-axis) at particular temperature. Panels **A–C** contain phenotypes of homozygous flies, whereas **A’–C’** are dedicated to heteroallelic combinations with *tim*^01^. Comparison of *τ* (shown as mean ± SEM) for all three mutants and wild-type (Canton strain) flies (wt-CS) for **(D)** homozygotes and **(D’)** heteroallelic combinations with *tim*^01^. **(E)** Alignment of mutant protein sequences with TIM from representative insects (see the text and legend of Figure 1 for more details on insect taxons). Mutation in position 270 (red box) possibly affects phosphorylation of serine residues in positions 268 and 274 (highlighted by red boxes). Only residues which phosphorylation is possibly affected by mutation are highlighted in the alignment of mutants and wt TIM proteins and prediction scores are indicated above the amino acid. The colors correspond to score prediction from NetPhos 3.1 (color coding is shown below the alignment).

Three lines with altered *τ* were recovered for the region 13 aa upstream from TIM^UL^ and we analyzed each of them in more detail (Table 4). Interestingly, all three included modification of tryptophan 270 (Figure 5E). The *tim^Δ*W270*^* deletion prolonged *τ* by ∼1.8 h and the single aa substitution *tim*^*W270Y*^ resulted in 0.6–1 h shortening of the free-running period. The effect was temperature independent in both cases and both lines were robustly rhythmic (>80% of rhythmic males at any temperature tested) (Figures 5A,A’,B,B’,D). In contrast, substitution of the tryptophan with three tyrosines (*tim*^*W270YYY*^) produced temperature-dependent lengthening of *τ* and severe arrhythmicity in homozygotes (see blue dots connected by line; scale on right *y*-axis); however, a comparable drop in rhythmicity was not observed in heteroallelic combinations with *tim*^01^ (Figures 5C,C’,D,D’). For statistical comparison of *τ* in homozygotes, heteroallelic combinations with *tim*^01^, and wild-type flies, see Supplementary Figure S4.

Furthermore, according to NetPhos 3.1 predictions, these mutations may impact the phosphorylation of the nearby serines in position 268 and 274, and although this needs to be experimentally tested, it presents an intriguing possible explanation of the functional significance of this region (Figure 5E). In the wild-type protein (TIM^wt^), serine 268 is not likely to be phosphorylated (score 0.47, below the threshold 0.5), whereas the W270 deletion raises the prediction score to 0.78 and substitution by three tyrosines (*tim*^*W270YYY*^) to 0.55. Similarly, serine at position 274 has a phosphorylation prediction score of 0.56, just above the threshold, but this is substantially increased by the presence of either *tim^Δ*W270*^*, *tim*^*W270Y*^, or *tim*^*W270YYY*^ (to 0.94, 0.82, or 0.92, respectively).

### Regions in Vicinity of TIM^rit^ and TIM^blind^ Mutations

**FIGURE 6:**
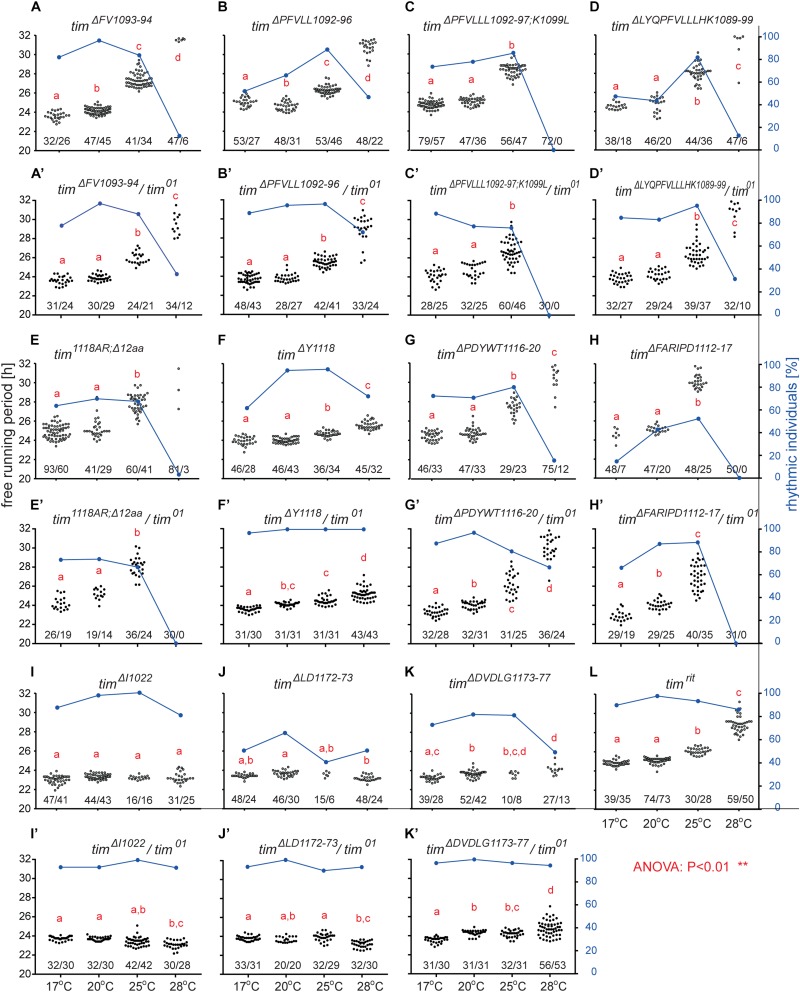
Mutants isolated in vicinity of *tim*^*rit*^ and *tim*^*blind*^. **(A–L)** Each dot represents *τ* of individual fly as was recorded during 10 days in DD and specified temperature. Lowercase red letters above dots indicate a significant difference between “a” and “b” and “c” and “d” (one-way ANOVA with Tukey–Kramer’s *post hoc* test, *P* < 0.01). Numbers below dots correspond to number of measured/rhythmic flies at particular temperature. Blue dots connected with line correspond to percentage of rhythmic individuals (right *y*-axis) at particular temperature.

**FIGURE 7:**
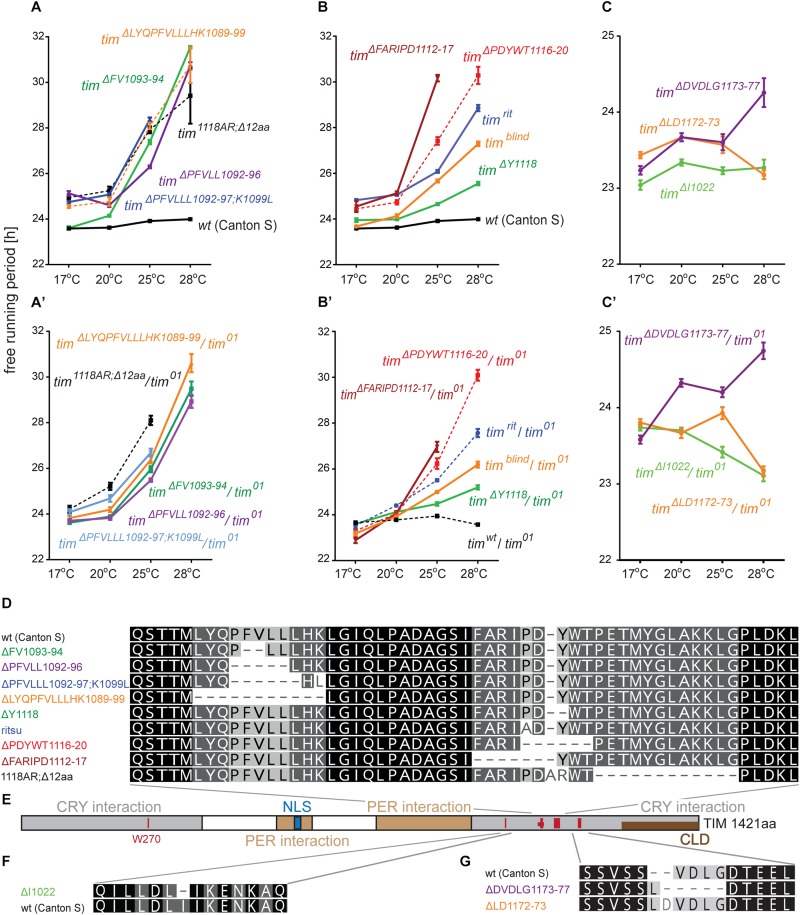
Summary of phenotypes for homozygous **(A–C)** and heteroallelic combinations with *tim*^01^ **(A’–C’)** mutants isolated in vicinity of *tim*^*rit*^ and *tim*^*blind*^. Name of each mutation is color-coded to ease orientation in the sequence alignments **(D,F,G)**. **(E)** Schematic depiction of TIM protein with position of mutated regions (red bars), nuclear localization signal (NLS), PERIOD interacting domain (PER interaction), CRYPTOCHROME interacting domain (CRY interaction), and cytoplasmic localization domain (CLD). Dashes (−) correspond to gaps in the alignment (which result from insertions or deletions). For statistical comparison between mutants and heteroallelic combinations, see Supplementary Figure S5.

**FIGURE 8:**
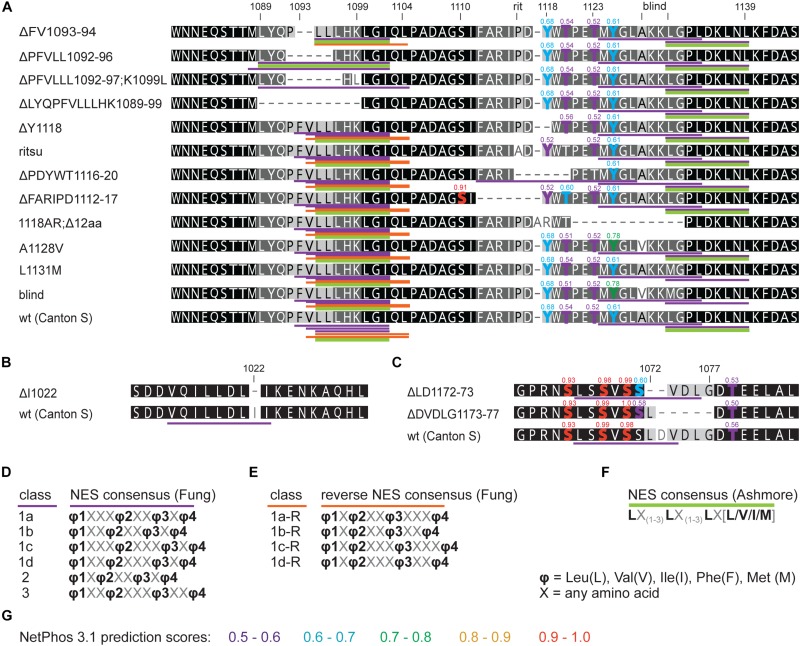
*in silico* prediction of nuclear export signals (NESs) and phosphorylation in *tim* mutants. Protein alignments of **(A)** *ritsu*, *blind*, and NES 1093-1104 regions, **(B)** NES 1015-1023, and **(C)** NES 1166-1174 with underlined NES motifs. Purple, orange, and green colors code for NES classes according to consensus shown in **D–F**. Residues which phosphorylation is possibly affected by mutation are highlighted in the alignment and scores are indicated above the amino acid. **(G)** The colors correspond to prediction from NetPhos 3.1.

An interspecific comparison of insect TIM proteins identified a conserved region near and especially upstream of the TIM^blind^ and TIM^rit^ mutations. This includes a putative NES overlapping with TIM^blind^ and a second putative NES, FVLLLHKLGIQL (residues 1093-1104), located 12-23 aa upstream of TIM^rit^. Both NESs were also predicted by the strict (“Ashmore”) and the less strict (“Fung”) consensus searches (Figure 2). Again, we probed the functional significance of this region by inducing NHEJ-mediated mutagenesis followed by locomotor activity screening. Four different mutants with abnormal *τ* were recovered for NES^1093–1104^ consisting of 2 to 11 aa long deletions (Figure 7D). In all four cases, *τ* gradually increased with rising temperature and was significantly longer compared to controls at 25°C and even more so at 28°C. For statistical comparison of *τ* in homozygotes, heteroallelic combinations with *tim*^01^, and wild-type flies, see Supplementary Figure S5. Furthermore, the percentage of rhythmicity was severely reduced in three of these mutants at 28°C with *tim^Δ1092–97:PFVLLL;K1099L^* being completely arrhythmic both as homozygotes and in heteroallelic combination with *tim*^01^ (Figures 6A–D,A’–D’). This strongly contrasts with the relatively robust rhythmicity (>75%) observed in all four mutants at 25°C. Detailed sequence analysis revealed various degrees of NES^1093–1104^ motif disruption in all four mutants (Figure 8A). Five overlapping NES classes (“Fung”) and one strict motif (“Ashmore’s”) can be found within the region 1093-1104 in wild-type TIM and all of them are completely lost in *tim^Δ*LYQPFVLLLHK1089–99*^*, while *tim^Δ1092–97:PFVLLL;K1099L^* has one putative “Fung’s” NES class remaining, while in *tim^Δ*FV1093–94*^* and *tim^Δ*PFVLL1092–96*^*, two NES^1093–1104^ according to “Fung” classes and one strict motif remain. Although all four NES^1093–1104^ mutants are phenotypically very similar, the degree of NES modification is quite different. The common feature of all mutants is the absence of the 1c-R class NES consensus (Figure 8A).

Another mutant completely removing the NES^1124–1139^, *tim^1118ARΔ12^*, overlaps with the TIM^blind^ mutations. However, this mutant also contains a substitution of tyrosine 1118 (a residue which is predicted to be phosphorylated) with alanine and arginine. Moreover, another predicted phosphorylation target, Y1125, is also missing in *tim^1118ARΔ12^* making it hard to interpret the relative importance of any of these sites for the observed phenotype (Figure 8). Similarly to the NES^1093–1104^ mutants, *tim^1118ARΔ12^* produces a longer *τ* at 25°C and severe (nearly complete) arrhythmicity at 28°C (Figures 6E,E’). We further addressed the role of the two TIM^blind^ residues and corresponding NES in the two re-engineered mutants. All predicted NES motifs near TIM^blind^ remained intact in TIM^A1128V^, yet this mutation produces mostly arrhythmic individuals and the few rhythmic flies show a potential temperature compensation defect (Figure 1E). In contrast, TIM^L1128M^ which affects the strict (“Ashmore”) NES motif is robustly rhythmic and perfectly temperature-compensated (Figure 1F). Notably, both the TIM^blind^ and TIM^A1128V^ mutations enhance the phosphorylation prediction score of Y1125 (Figure 8A). However, it is unknown, if Y1125 is phosphorylated *in vivo*.

Several *τ*-altering mutants mapping close to the TIM^rit^ mutation were recovered (Figure 7D). The most severe phenotype was seen in *tim^Δ*FARIPD1112–1117*^* (Figures 6H,H’, 7B,B’) with overall low rhythmicity across temperatures and complete arrhythmicity at 28°C. Free running period is ∼30 h at 25°C for homozygotes, the longest *τ* of all mutants recovered in this region. However, *τ* was significantly shorter (∼27 h) in heteroallelic combinations with *tim*^01^ at 25°C (Supplementary Figure S5). A slightly less severe impact on rhythmicity was found in *tim^Δ*PDYWT1116–20*^*, which is rhythmic at all temperatures, although rhythmicity at 28°C drops below 20% in homozygotes, whereas two-third of heteroallelic combinations with *tim*^01^ remain rhythmic (Figures 6G,G’). The flies also display loss of temperature compensation as their free-running period gets longer at high temperatures. For comparison, the proline 1116 to alanine substitution in *tim*^*rit*^ produces gradually longer *τ* at 25 and 28°C with rhythmicity above 80% at all temperatures tested (Figures 6I,J,L). The smallest, yet significant extension of *τ* was observed in *tim^Δ*Y1118*^* (Figure 6F), a mutant lacking tyrosine 1118, which is likely phosphorylated (score 0.68, Figure 8A). Phosphorylation score of Y1118 is reduced in TIM^rit^ to 0.52 and this residue is deleted, together with the surrounding four amino acids, in *tim^Δ*PDYWT1116–20*^*. The mutant with the strongest phenotype, *tim^Δ*FARIPD1112–1117*^*, has a lower predicted phosphorylation score for Y1118, identical with the score calculated in TIM^rit^. In addition, deletion of residues 1112-1117 (FARIPD) also changes the phosphorylation score of the upstream S1110 from a non-significant level to 0.91.

Systematic targeting of the TIM^rit^ and TIM^*blind*^ region and the conserved region 12-23 aa upstream of TIM^rit^ encompassing a putative NES^1093–1104^ motifs (FVLLLHKLGIQL) resulted in mutants showing various degrees of arrhythmicity and temperature-dependent *τ* increase. To elucidate whether the above-mentioned region including TIM^rit^, TIM^*blind*^, and NES^1093–1104^ is uniquely important for temperature compensation of the circadian clock, comparable mutagenesis was performed in regions with NES motifs located ∼50 aa downstream (NES^1166–1174^) and ∼50 aa upstream (NES^1015–1023^). The mutagenesis was successful in both regions, which is demonstrated by our ability to isolate 9 and 8 fully arrhythmic mutant lines, respectively (Table 3). In contrast, only three rhythmic mutants with altered rhythmicity or changed *τ* were found and although one of them, *tim^Δ*DVDLG1173–77*^*, produces significant lengthening of *τ* with rising temperature, the change is minimal, about 1 h (Figures 6I,J,K,K’,L, 7C,C’,E–G). The deletion in *tim^Δ*DVDLG1173–77*^* destroys the putative NES^1166–1174^ motif and slightly changes the phosphorylation score of S1071 (from 0.49 to 0.58) and T1079 (from 0.56 to 0.50) (Figure 8C). A partially overlapping deletion, *tim^Δ*LD1172–73*^*, does not remove the NES^1166–1174^, but the phosphorylation scores are similarly affected for both S1071 (from 0.49 to 0.60) and T1079 (from 0.56 to 0.53). This mutant is characterized by a minimal shortening of *τ* at 28°C (0.8 h shorter than wt, Supplementary Figure S5J). Finally, the *tim^Δ*I1022*^* mutant produces a 0.3–0.75 h shorter *τ* than wt flies at all temperatures (Figures 7C,C’ and Supplementary Figure S5I). This single amino acid deletion removes the NES^1015–1023^ motif (Figure 8B).

### Immunocytochemistry

**FIGURE 9:**
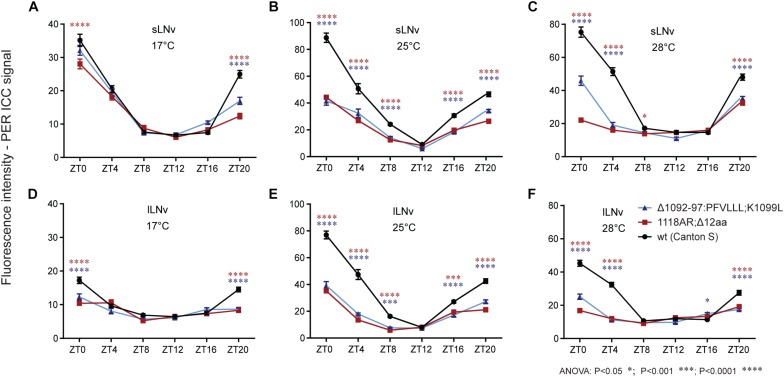
Immuno-localization of PER in small and large ventrolateral neurons (s-LNv, l-LNv). Relative quantifications show role of **(A–F)** temperature on PER immunoreactivity within genotype. Only statistical difference between wt and mutants is shown for each ZT. For differences, see Supplementary Figure S6. s-LNv and l-LNv were plotted separately.

In order to elucidate the impact of the newly isolated *tim* mutations on the temporal clock protein expression pattern in the circadian clock neurons, we performed ICC on whole mount fly brains. Since no TIM antibody was available, we used PER immunostainings as a proxy to visualize the progression of the PER-TIM negative feedback loop. Two mutants covering the *rit* (*tim^Δ1092–97:PFVLLL;K1099L^*) and *blind* (*tim^1118ARΔ12^*) region were selected. Both mutants show relatively robust rhythmicity at 17–25°C, but are completely arrhythmic at 28°C (Figures 6C,E). In both, wt and mutants, the PER level was cycling during the day at every temperature, but in mutants, the amplitude of the oscillation was reduced (Figure 9 and Supplementary Figure S6). At 17°C, PER intensity was almost comparable in small ventrolateral (s-LNv) neurons between wt, *tim^Δ1092–97:PFVLLL;K1099L^*, and *tim^1118ARΔ12^*, with a peak at ZT0 and a trough around ZT12. A similar trend was observed in the large ventrolateral (l-LNv) neurons, with the difference that the relative staining intensity was about half the intensity detected in s-LNv (Figure 9D). At 25°C, a clear difference in the intensity of the PER signal was observed between wt and the two *tim* mutants in both s-LNv and l-LNv in all ZTs with the exception of ZT12 (Figures 9B,E and Supplementary Figures S6B,C). At 28°C, the highest level of PER was detected in wt, lower levels in *tim^Δ1092–97:PFVLLL;K1099L^* and the lowest level was detected in *tim^1118ARΔ12^* at ZT0, ZT4, and ZT20 (Figures 9C,F, Supplementary Figure S7). The pattern of immunostaining intensity at 17 and 25°C is thus consistent with the locomotor activity phenotypes observed in the mutants. However, both mutants are virtually arrhythmic at 28°C, yet the expression levels of PER were clearly different between them at the beginning of the day and at the end of the night.

The experimental set-up allowed us to perform semi-quantitative comparisons between temperatures within each genotype. In s-LNv neurons of wt flies, the lowest signal intensities were observed at 17°C, whereas expression levels were more or less comparable at 25 and 28°C. In l-LNv neurons, signal intensities increased with temperature: the lowest expression was observed at 17°C, intermediate levels at 28°C, and the maximal expression at 25°C. In both mutants, expression levels varied much less across temperatures due to lower levels detected at 25 and 28°C.

## Discussion

### Targeted Screen for Temperature Compensation Mutants

Circadian clock research is a remarkably successful field of experimental biology and the molecular mechanisms that build up circadian oscillators are well understood, especially in *Drosophila*. Despite these huge successes, there are a number of distressingly large gaps in our understanding of biological timekeeping. One of the key features of circadian clocks is their ability to keep a largely unchanged pace regardless of temperature, a phenomenon termed temperature compensation. To specifically generate new mutants with temperature compensation defects, we utilize the efficacy of the CRISPR/CAS9 technology as a tool for targeted mutagenesis. Simple genetic crosses are used to establish homozygous mutants and their *τ* is determined in a temperature step-up protocol to specifically identify mutants even if they only exhibit very subtle impairments in temperature compensation.

Although the position where the CAS9 protein cleaves chromosomal DNA is defined by the gRNA sequence, the actual mutations resulting from subsequent NHEJ are variable and virtually unpredictable in/dels. As a consequence, various degrees of gradual amino acid deletions (see Figures 5E, 7D) and phenotypic changes (see Figures 7A–C) were obtained. More than one-quarter of created lines were circadian mutants, but only 3% of screened lines were unique mutants with altered *τ*. Obviously, a higher percentage of mutants was probably induced, but if the phenotypic change was below our recognition, these lines were discarded. Yet, the success rate is remarkably higher than in EMS screens and importantly, identification of mutation is straightforward, fast, and cheap, compared to time demanding mapping after classical mutagenesis. The obvious limitation is that the screen presented here is strictly hypothesis-driven (Curtiz and Wallis, 1942) and mutations are created only in candidate regions of already established circadian clock genes. Therefore, this approach is suitable to saturate genes with targeted mutations and to assess the function of specific regions including coding sequence or cis-regulatory motifs in promoters.

### *Timeless* in Temperature Compensation of the Circadian Clock

This study exploited TIM in selected insect representatives. Despite the long history from the common insect ancestor (>400 million years ago), some regions of TIM are well conserved across insect species, pointing to their possible functional significance. Therefore, we experimentally tested the role of eight conserved motifs by targeted mutagenesis and isolated circadian clock mutants in seven of them. Three remarkably conserved regions located closely together, TIM^rit^, TIM^*blind*^, and NES^1093–1104^, were functionally identified as particularly critical for temperature compensation. The importance of nuclear export for proper function of TIM is well established (Ashmore et al., 2003) and *tim*^*blind*^ overlaps with NES^1131–1139^ (Wülbeck et al., 2005). It is surprising that the two amino-acid replacements encoded by *tim*^*blind*^ lead to a strong temperature compensation phenotype, because both the alanine to valine substitution at position 1128 and the leucine to methionine change at position 1131 are conservative replacements. Even more surprising is the strong impact on clock function observed in the single mutant *tim*^*A1128V*^. The few rhythmic *tim*^*A1128V*^ individuals are associated with variable periods but do exhibit potential period lengthening with increasing temperatures (Figures 1E,H and Table 5). It is therefore possible that in the *tim*^*blind*^ double-mutant, the L1131M substitution, which shows no phenotype on its own, somehow suppresses the A1128V substitution, resulting in restauration of rhythmicity but maintenance of the temperature compensation phenotype. It would be very interesting to see, whether TIM^blind^ residues play a similar role in *P. apterus* TIM, where neither “Ashmore’s” nor “Fung’s” NESs are predicted (Figure 2A). This type of experiment might be possible in future, as genome editing slowly becomes accessible even in non-model insects including *P. apterus* (Kotwica-Rolinska et al., 2019).

The NES^1093–1104^ region described here consists of three forward and two reverses NES consensuses. However, comparably strong temperature compensation defects were observed in all mutants targeting NES^1093–1104^, although different numbers of NES consensuses were depleted. Either 1c-R NES is the only essential export signal, or residues 1089-1099 have some additional role for TIM structure. Notably, all three mutants in the *tim*^*rit*^ region mapping just 8–14 amino acids downstream from NES^1093–1104^ show a temperature compensation defect, although none of these mutations directly affects NES^1093–1104^. Additionally, neither mutation in NES^1015–1023^ nor in NES^1166–1174^ had an impact on temperature compensation. Currently, it is therefore unclear if nuclear export is indeed important for temperature compensation, or if other alterations (e.g., changes in phosphorylation), or a combination of both, contribute to the temperature compensation phenotypes observed in several of the mutants described in this study. Analysis of the detailed subcellular localization of the mutated TIM proteins will hopefully point to the possible mechanism contributing to temperature compensation.

Our comparison often revealed a shorter *τ* in heteroallelic combinations of various mutants with *tim^01^*, a combination with only one partially functional *tim* copy, when compared to homozygous mutants (Supplementary Figure S5). This contrasts with the dose-independent role of wild-type TIM reported previously at 25°C (Rothenfluh et al., 2000a, b; Ashmore et al., 2003) and our results obtained for TIM^wt^ at all four tested temperatures (Supplementary Figure S5A). Notably, a ∼1 h shorter *τ* was also observed for *tim^*rit*^/tim^01^* heterozygotes exposed to different temperatures (Matsumoto et al., 1999) supporting the possibility that in specific temperature conditions, the amount of a mutated TIM protein might be important for *τ*.

Our ICC data indicate that the PER immunostaining signal is strongly affected by *tim* mutations and that the low PER signal is more profound at high temperatures. Although the ICC data were obtained during LD, whereas circadian phenotypes were recorded in DD, PER immunostaining intensities are consistent with the behavioral defects of particular mutants. It is not clear if the stability of mutant TIM is primarily affected at 28°C, or if the interaction between PER and TIM is somehow influenced by the mutations. Therefore, PER immunostaining only serves as a proxy for the status of the PER-TIM negative feedback loop. In this regard, it is worth noting that PER and TIM do not co-localize perfectly in various neurons during the circadian cycle (Shafer et al., 2002; Wülbeck et al., 2005). Moreover, TIM also interacts with CRY in a light-dependent manner (Ceriani et al., 1999) and thus the here newly induced mutations may also affect this interaction. However, the possible change in CRY-TIM interaction should not impact free running period in DD, as CRY-depleted flies have a normal *τ* (Stanewsky et al., 1998; Dolezelova et al., 2007). The light resetting capacity of the new *tim* mutants is currently unknown, but given the light entrainment phenotype of *tim*^*blind*^ connected with the partial resistance of TIM^blind^ to light-induced degradation, it is possible that at least some of the new alleles are affected (Wülbeck et al., 2005).

The subcellular localization of TIM is connected with its phosphorylation. Long *τ tim* mutants are characterized by hypophosphorylated TIM constrained in the cytoplasm as it is known that interaction with the nuclear transport machinery is dependent on the phosphorylation state of TIM (Jang et al., 2015). Likewise, TIM^BLIND^ is hypophosporylated at all times during the circadian cycle and accumulates in the cytoplasm of photoreceptor cells and LNv clock neurons, with only minor effects on PER phosphorylation and subcellular localization (Wülbeck et al., 2005). Indeed, several of our mutants between positions 1112 and 1120 affect phosphorylation predictions without altering the NES sequence. For example, deletion of tyrosine 1118 produces mild and gradual lengthening of *τ* at 25 and 28°C. Interestingly, a more pronounced *τ* extension is observed in *tim*^*rit*^ mutants even though its 1116 proline to alanine substitution only reduces the phosphorylation prediction score for tyrosine 1118. The *tim^Δ*PDYWT1116–20*^* mutation results in an even longer *τ* combined with substantially reduced rhythmicity at 28°C. The most severe phenotype with complete arrhythmicity at 28°C, generally low rhythmicity, and remarkable *τ* extension at 25°C is observed in *tim^Δ*FARIPD1112–17*^* mutants. Interestingly, deletion of FARIPD changes the phosphorylation prediction score for serine 1110 (from a non-significant score to 0.91) in addition to mild changes in scores for tyrosine 1118 and threonine 1120. Although further functional experiments are needed to determine the actual impact of the novel mutations on TIM localization and phosphorylation, the collection of mutants presented here points to a new region of TIM important for temperature compensation.

## Data Availability Statement

All datasets generated and analyzed for this study are included in the article/Supplementary Material.

## Author Contributions

SS and DD designed the study. SS performed majority of NHEJ experiments, analyzed, and interpreted the results. SF combined *per*, *tim*, and *cry* alleles and assessed their phenotypes. RS and SF independently observed the temperature compensation phenotype associated with *tim*^*blind*^. AG performed HDR reengineering of *tim*^*blind*^ mutants and their behavioral analysis. MD performed immunohistochemical experiments. GM contributed gRNA design and participated in early steps of the screen. DD supervised the study and together with SF wrote the manuscript with input from all co-authors.

## Conflict of Interest

The authors declare that the research was conducted in the absence of any commercial or financial relationships that could be construed as a potential conflict of interest.

## Footnotes

1 https://flycrispr.org/target-finder/
