# Supplementary material for: New Drosophila Circadian Clock Mutants Affecting Temperature Compensation Induced by Targeted Mutagenesis of Timeless
Source: Front Physiol. 2019 Dec 3;10:1442. doi: 10.3389/fphys.2019.01442 (PMC6901700; doi:10.3389/fphys.2019.01442)
Supplement: Supplementary file 1 [file Data_Sheet_1.docx]

Captions for the Supplementary Figures S1-S7

**Supplementary Figures S1|** Scheme of genetic crosses used to (**A**) obtain gRNA-expressing line, and (**B**) to initiate NHEJ by combining gRNA-expressing line with CAS9-expressing line. The ‘TBX’ number correspond to Kondo and Ueda (2013).

**Supplementary Figures S2**|(**A**)Free running period and (**B**) detailed genotypes of lines used in the current study (names in blue in panel A) and lines that might be used for similar experiments.

**Supplementary Figures S3**| **(A, B, C)** DNA and protein sequences of obtained mutants compared to control (*wt*, *wild type*)

**Supplementary Figures S4**| Statistical comparison of the free running periods for mutants near the *ultralong* region of *timeless.*

**Supplementary Figures S5**| Statistical comparison of the free running periods for mutants near the *blind* and *ritsu* regions of *timeless.*

**Supplementary Figures S6**| Statistical comparison of the PER staining intensity between three mutants at three temperatures.

**Supplementary Figures S7**| Examples of PER stainings at ZT0 (=light on) at three temperatures.
