# Supplementary figures and images for: New Drosophila Circadian Clock Mutants Affecting Temperature Compensation Induced by Targeted Mutagenesis of Timeless
Source: Front Physiol. 2019 Dec 3;10:1442. doi: 10.3389/fphys.2019.01442 (PMC6901700; doi:10.3389/fphys.2019.01442)

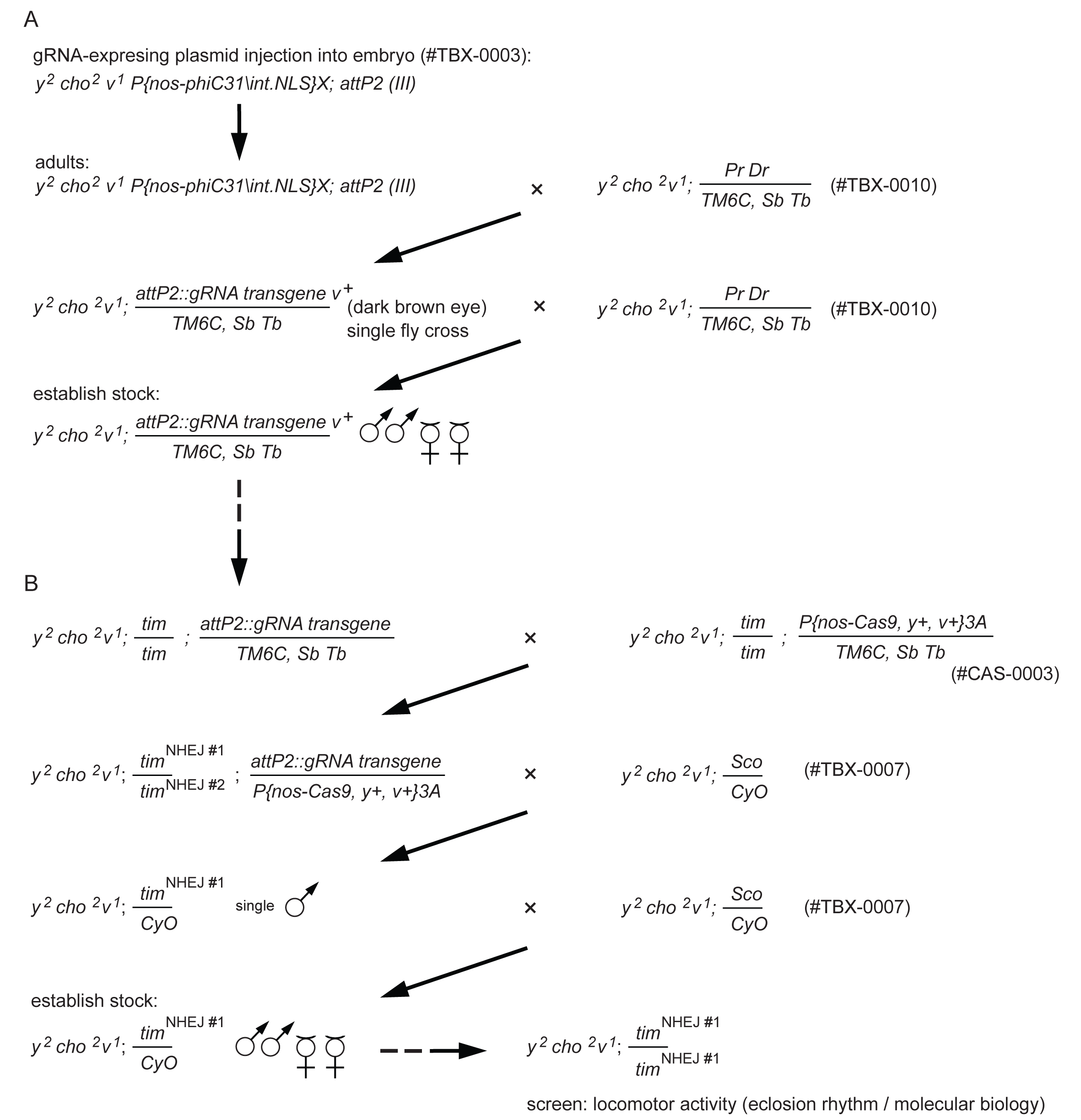

Supplement: Supplementary file 2 [file Image_1.tif]

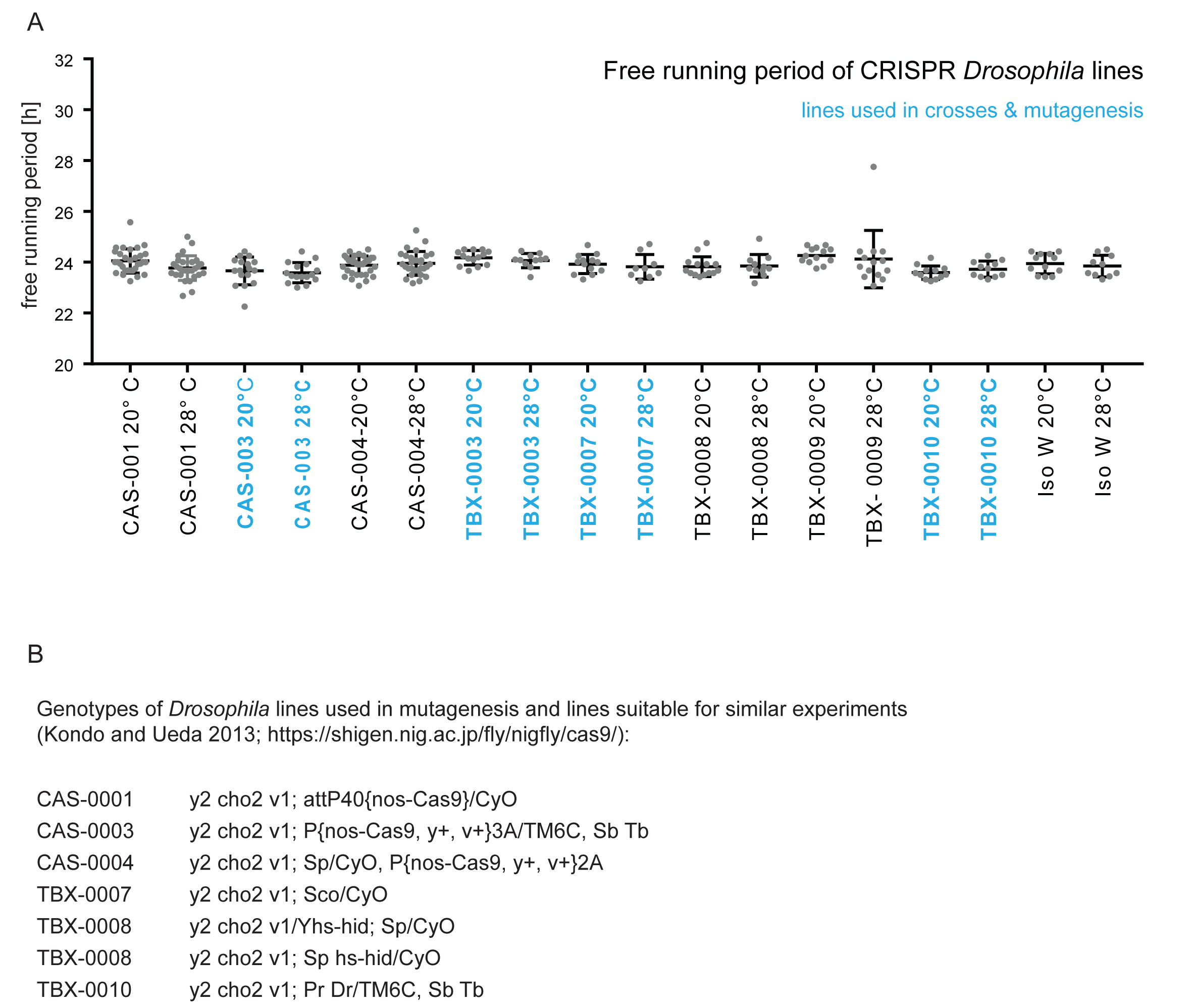

Supplement: Supplementary file 3 [file Image_2.tif]

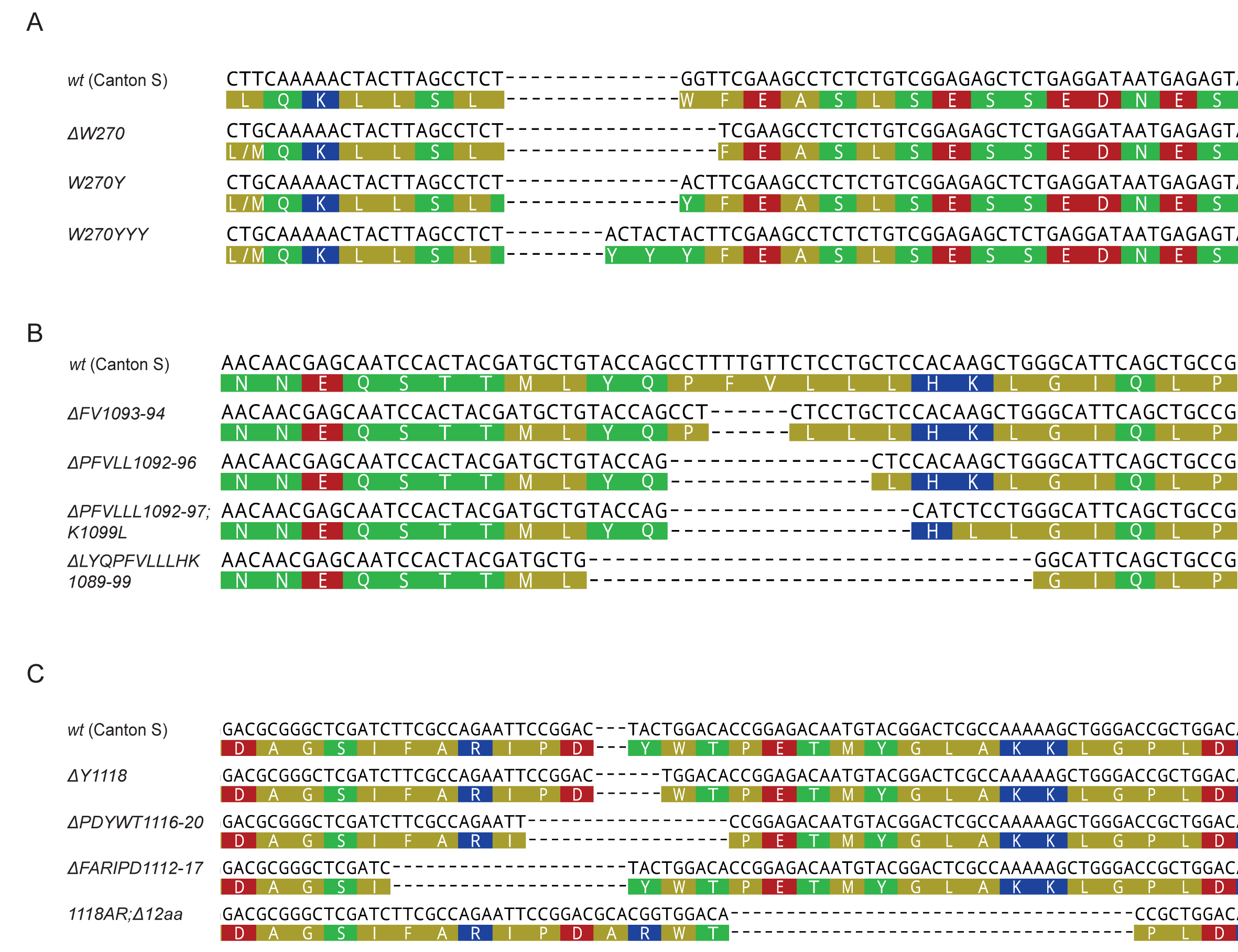

Supplement: Supplementary file 4 [file Image_3.tif]

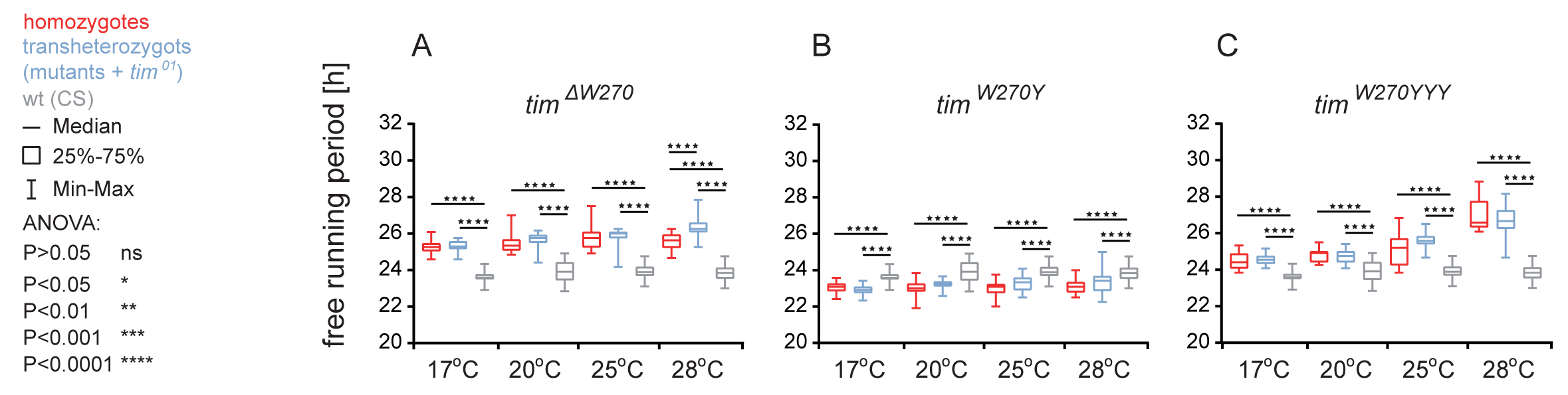

Supplement: Supplementary file 5 [file Image_4.tif]

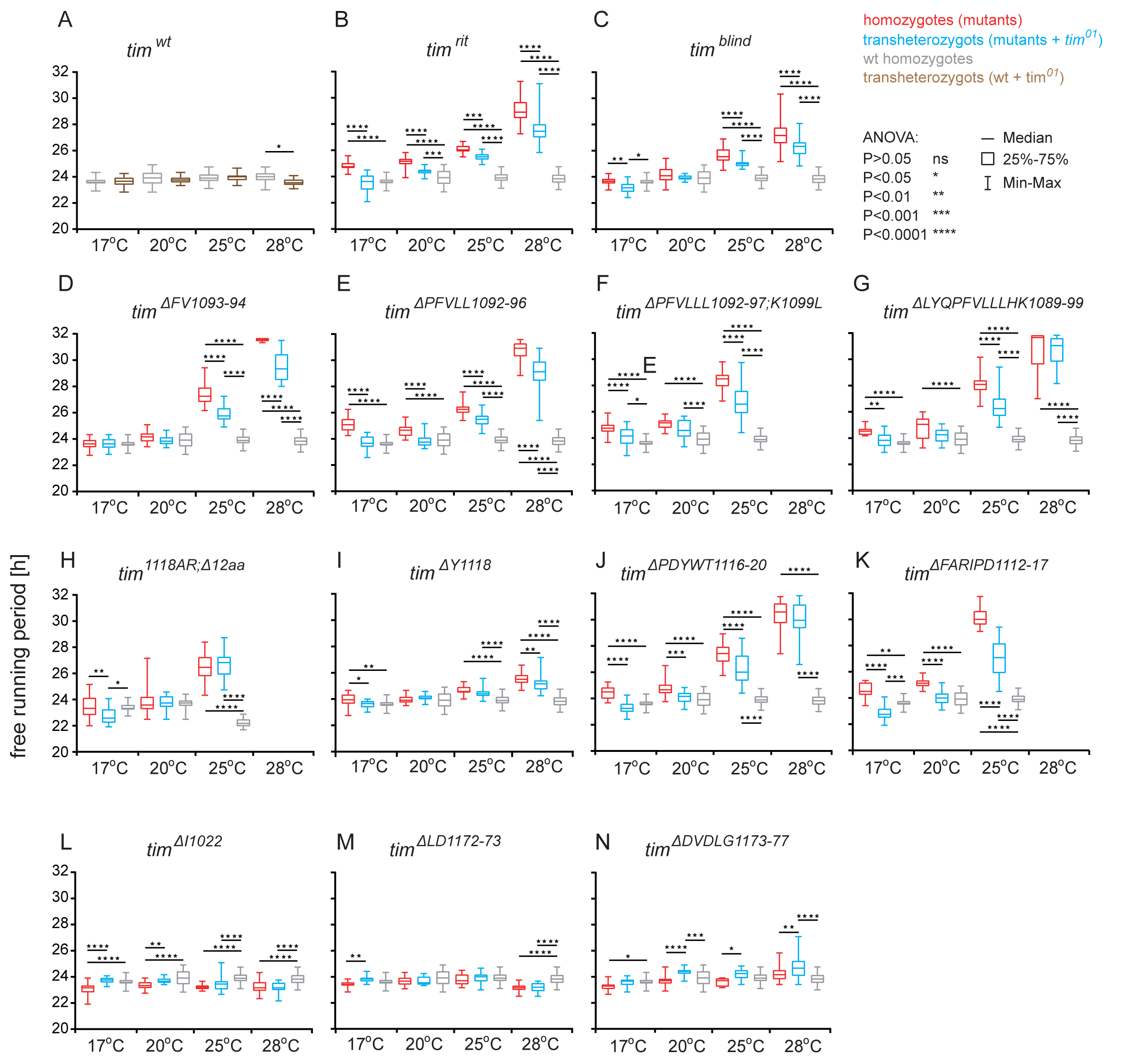

Supplement: Supplementary file 6 [file Image_5.tif]

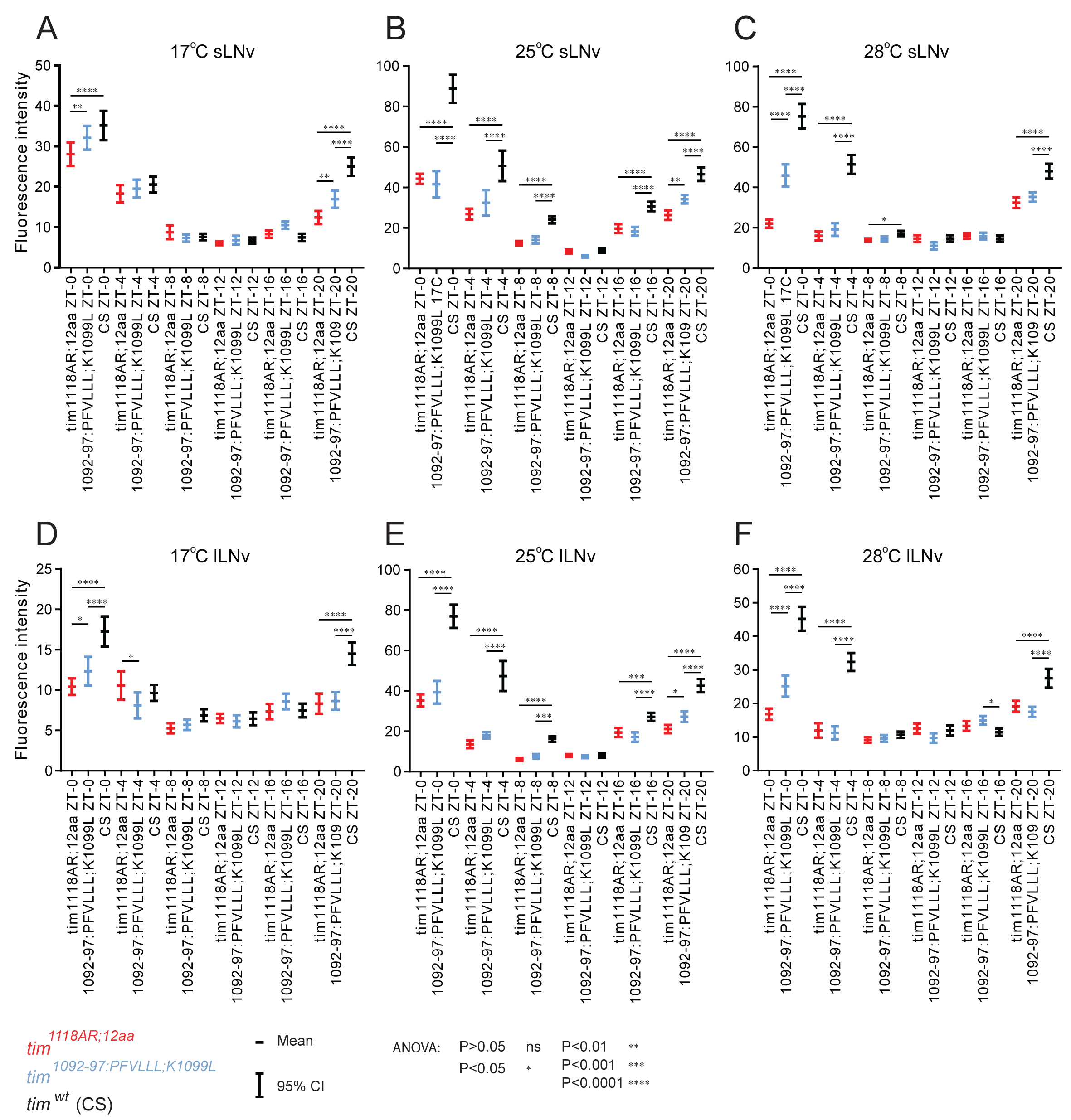

Supplement: Supplementary file 7 [file Image_6.tif]

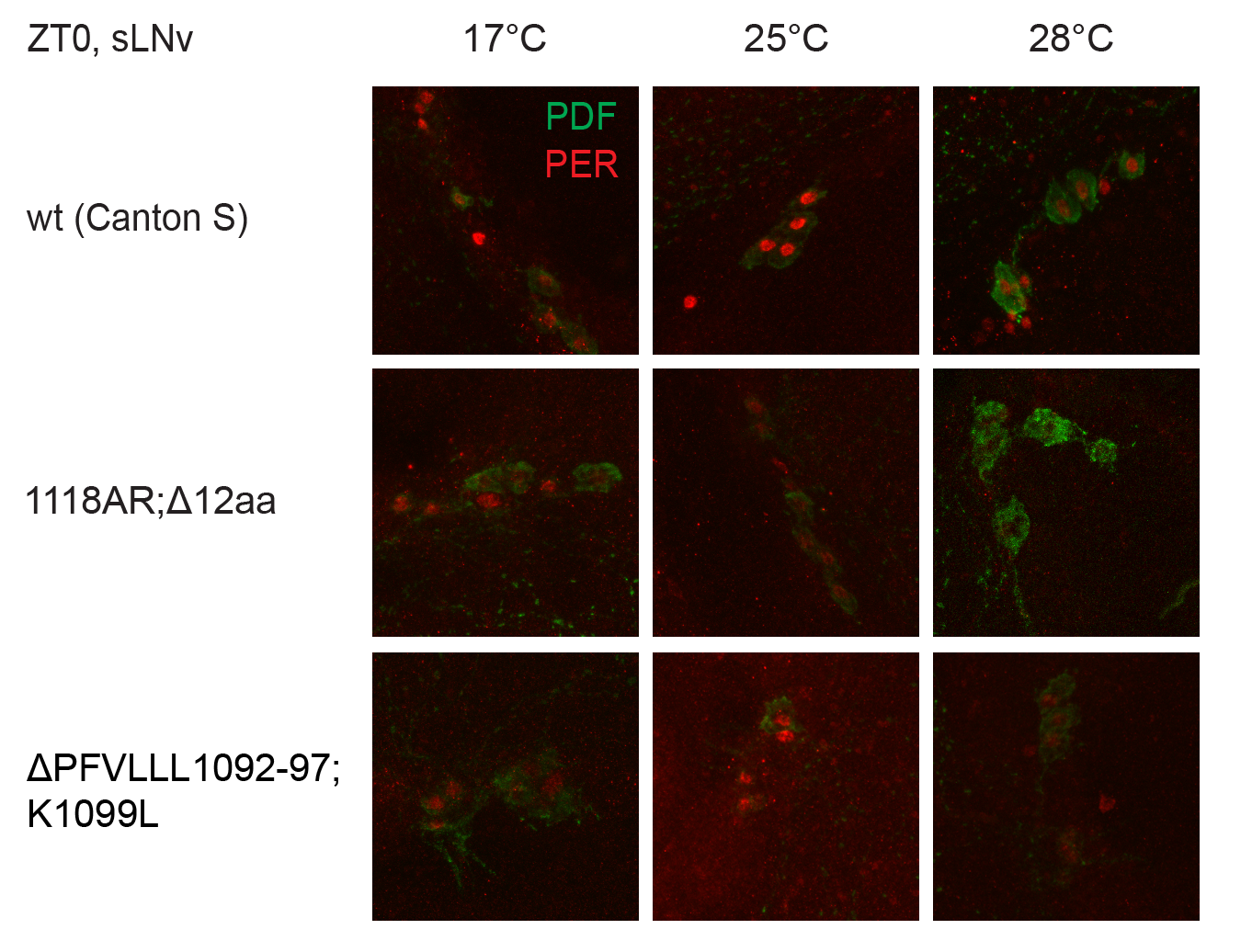

Supplement: Supplementary file 8 [file Image_7.tif]
