# Supplementary material for: New Drosophila Circadian Clock Mutants Affecting Temperature Compensation Induced by Targeted Mutagenesis of Timeless
Source: Front Physiol. 2019 Dec 3;10:1442. doi: 10.3389/fphys.2019.01442 (PMC6901700; doi:10.3389/fphys.2019.01442)
Supplement: Supplementary file 9 [file Table_1.DOC]

| **Table S1. part A Free-running locomotion in DD at different temperatures** | | | | | | | | | |
| --- | --- | --- | --- | --- | --- | --- | --- | --- | --- |
| genotype | 18°C | | | 25°C | | | 28°C | | |
|  | *N* rhythmic (%) | FRP (+/-) [hrs] | *N* non-rhythmic (%) | *N* rhythmic (%) | FRP (+/-) [hrs] | *N* non-rhythmic (%) | *N* rhythmic (%) | FRP (+/-) [hrs] | *N* non-rhythmic (%) |
| *tim^blind^;cry^01^* | 51 (84) | 24 (+1/-1) | 10 (16) | 54 (96) | 25 (+0.5/-0.5) | 2 (4) | 59 (98) | 26 (+1/-1.5) | 1 (2) |
| *tim^blind^;cry^02^* | 26 (87) | 24 (+1/-1) | 4 (13) | 25 (96) | 25 (+1/-1) | 1 (4) | 31 (97) | 26 (+2/-0.5) | 1 (3) |
| *tim^blind^;cry^03^* | 28 (93) | 24 (+1/-1) | 2 (7) | 28 (97) | 25.5 (+1/-0.5) | 1 (3) | 28 (97) | 26.5 (+2/-1.5) | 1 (3) |
| *tim^blind^;cry^b^* | 20 (87) | 24 (+1.5/-0.5) | 3 (13) | 26 (100) | 25.5 (+1/-1.5) | 0 (0) | 31 (100) | 26.5 (+1.5/-1) | 0 (0) |
| *tim^blind^;cry^m^* | 16 (80) | 24 (+2.5/-1) | 4 (20) | 29 (94) | 26 (+0.5/-0.5) | 2 (6) | 28 (93) | 27 (+1.5/-0.5) | 2 (7) |
| *tim^blind^;Sb/TM6B* | 21 (95) | 23.5 (+1/-1) | 1 (5) | 29 (100) | 25 (+1.5/-0.5) | 0 (0) | 24 (100) | 26 (+2/-0) | 0 (0) |
| *tim^rit^;cry^01^* | 23 (100) | 25 (+1/-1) | 0 (0) | 26 (93) | 26.5 (+1.5/-1.5) | 2 (7) | 18 (86) | 28 (+2.5/-2) | 3 (14) |
| *tim^rit^;cry^02^* | 23 (100) | 24.5 (+1/-1) | 0 (0) | 28 (93) | 26.5 (+2/-1.5) | 2 (7) | 19 (83) | 30 (+3.5/-3.5) | 4 (7) |
| *tim^rit^;cry^03^* | 24 (100) | 25 (+1/-2) | 0 (0) | 25 (86) | 28 (+1.5/-2.5) | 4 (14) | 14 (88) | 31.5 (+2/-2.5) | 2 (12) |
| *tim^rit^;cry^b^* | 22 (96) | 24.5 (+0.5/-1) | 1 (4) | 26 (96) | 26.5 (+3/-1) | 1 (4) | 17 (85) | 31 (+3.5/-3) | 3 (15) |
| *tim^rit^;cry^m^* | 21 (100) | 24.5 (+0.5/-0.5) | 0 (0) | 31 (100) | 27.5 (+1.5/-1.5) | 0 (0) | 12 (63) | 30 (+3.5/-3.5) | 7 (37) |
| *tim^rit^;Sb/TM6B* | 16 (100) | 25 (+0.5/-0.5) | 0 (0) | 31 (100) | 26.5 (+1.5/-1.5) | 0 (0) | 15 (94) | 28.5 (+2.5/-0.5) | 1 (6) |
| *tim^S1^;cry^01^* | 7 (30) | 20.5 (+0.5/-0.7) | 16 (70) | 18 (64) | 20 (+1/-1.5) | 10 (36) | 16 (70) | 19.5 (+2.5/-1) | 7 (30) |
| *tim^S1^;cry^02^* | 3 (14) | 21 (+1.5/-0.5) | 18 (86) | 13 (54) | 20.5 (+1/-0.8) | 11 (46) | 14 (70) | 19.7 (+0.3/-0.7) | 6 (30) |
| *tim^S1^;cry^03^* | 18 (75) | 21.5 (+0.5/-1.5) | 6 (25) | 25 (86) | 21.5 (+1/-2) | 4 (14) | 18 (100) | 20 (+1/-2) | 0 (0) |
| *tim^S1^;cry^b^* | 12 (52) | 20.5 (+1/-0.5) | 11 (48) | 22 (79) | 20 (+2.5/-1.5) | 6 (21) | 17 (89) | 19.7 (+1.8/-1.2) | 2 (11) |
| *tim^S1^;cry^m^* | 13 (57) | 21 (+0.5/-1) | 10 (43) | 21 (95) | 21 (+1/-1.5) | 1 (5) | 21 (91) | 21 (+0.5/-1) | 2 (9) |
| *tim^S1^;Sb/TM6B* | 18 (78) | 21 (+1/-2) | 5 (22) | 26 (97) | 21 (+1/-2) | 2 (3) | 16 (100) | 20.5 (+1/-0.5) | 0 (0) |
| *tim^L1^;cry^01^* | 19 (83) | 26.5 (+1/-2) | 4 (17) | 11 (92) | 26 (+1/-0.5) | 1 (8) | 10 (100) | 25 (+2/-0.5) | 0 (0) |
| *tim^L1^;cry^02^* | 9 (64) | 27 (+1/-1) | 5 (36) | 5 (83) | 27 (+0.5/-1) | 1 (17) | 10 (100) | 26 (+1/-0.5) | 0 (0) |
| *tim^L1^;cry^03^* | 20 (91) | 26 (+1/-0.5) | 2 (9) | 17 (89) | 27 (+0.5/-1) | 2 (11) | 16 (80) | 26 (+1/-0.5) | 4 (20) |
| *tim^L1^;cry^b^* | 6 (86) | 26.5 (+0.5/-1) | 1 (14) | 1 |  |  | 11 (100) | 25.5 (+0.5/-1.5) | 0 (0) |
| *tim^L1^;cry^m^* | 10 (77) | 26.25 (+0.25/-1.25) | 3 (23) | 24 (100) | 27.5 (+1/-2) | 0 (0) | 21 (100) | 26 (+1/-0) | 0 (0) |
| *tim^L1^;Sb/TM6B* | 7 (100) | 25 (+1/-0.5) | 0 (0) | 11 (85) | 26 (+1/-1.5) | 2 (15) | 8 (100) | 25.75 (+0.25/-0.25) | 0 (0) |

| **Table S1. part B Free-running locomotion in DD at different temperatures** | | | | | | | | | |
| --- | --- | --- | --- | --- | --- | --- | --- | --- | --- |
| Genotype | 18°C | | | 25°C | | | 28°C | | |
|  | *N* rhythmic (%) | FRP (+/-) [hrs] | *N* non-rhythmic (%) | *N* rhythmic (%) | FRP (+/-) [hrs] | *N* non-rhythmic (%) | *N* rhythmic (%) | FRP (+/-) [hrs] | *N* non-rhythmic (%) |
| *tim^UL^;cry^01^* | 16 (59) | 32 (+1.5/-3.5) | 11 (41) | 6 (25) | 33.25 (+2.25/-2.75) | 18 (75) | 12 (44) | 33 (+1.5/-0.5) | 15 (56) |
| *tim^UL^;cry^02^* | 14 (47) | 32 (+1.5/-3.5) | 16 (53) | 7 (32) | 35 (+0.5/-2) | 15 (78) | 11 (55) | 34.5 (+1/-1) | 9 (45) |
| *tim^UL^;cry^03^* | 15 (63) | 33 (+2/-3.5) | 9 (37) | 9 (41) | 34 (+1.5/-1) | 13 (59) | 9 (43) | 34 (+1/-1.5) | 12 (57) |
| *tim^UL^;cry^b^* | 22 (79) | 31.5 (+1.5/-2) | 6 (21) | 11 (69) | 33 (+1/-0.5) | 7 (31) | 17 (65) | 33.5 (+1/-1) | 9 (35) |
| *tim^UL^;cry^m^* | 35 (81) | 30 (+1.5/-1.5) | 8 (19) | 39 (72) | 32 (+2/-3) | 15 (28) | 50 (85) | 32.5 (+1.5/-1.5) | 9 (15) |
| *tim^UL^;Sb/TM6B* | 16 (100) | 30.75 (+2.25/-1.75) | 0 (0) | 15 (75) | 30 (+2.5/-2) | 5 (25) | 13 (62) | 33 (+1/-0.5) | 8 (38) |
| *per^SLIH^;Sp/CyO; cry^01^* | 21 (95) | 28 (+1/-2) | 1 (5) | 20 (83) | 27 (+1./-1) | 4 (17) | 10 (71) | 25.5 (+1/-0.5) | 4 (28) |
| *per^SLIH^;Sp/CyO; cry^02^* | 21 (91) | 27.5 (+1.5/-1) | 2 (9) | 24 (89) | 27 (+1.5/-1) | 3 (11) | 14 (70) | 26 (+1/-0.5) | 6 (30) |
| *per^SLIH^;Sp/CyO; cry^03^* | 21 (95) | 28.5 (+1/-2) | 1 (5) | 23 (92) | 28 (+1.5/-1.2) | 2 (8) | 19 (95) | 27 (+0.5/-1) | 1 (5) |
| *per^SLIH^;Sp/CyO; cry^b^* | 21 (95) | 28 (+1/-0.5) | 1 (5) | 20 (91) | 27 (+0.5./-1) | 2 (9) | 17 (100) | 26 (+0.5/-0) | 0 (0) |
| *per^SLIH^;Sp/CyO; cry^m^* | 23 (100) | 28.5 (+1/-1) | 0 (0) | 29 (97) | 28 (+0.5/-1) | 1 (3) | 22 (100) | 26.5 (+1.5/-0.5) | 0 (0) |
| *per^SLIH^;Sp/CyO; Sb/TM6B* | 20 (95) | 28 (+1/-1.5) | 1 (5) | 24 (92) | 27.5 (+0.5/-1) | 2 (8) | 14 (100) | 26 (+1.5/-0) | 0 (0) |
| *per^T^;Sp/CyO; cry^01^* | 16 (73) | 16.5 (+0.5/-0.5) | 6 (27) | 19 (66) | 16 (+1/-0.5) | 10 (34) | 6 (38) | 15.5 (+0.8/-0.5) | 10 (62) |
| *per^T^;Sp/CyO; cry^02^* | 17 (74) | 16.3 (+0.7/-0.8) | 6 (26) | 10 (71) | 16.15 (+0.85/-0.85) | 4 (29) | 4 (44) | 15.5 (+1.15/-0.35) | 5 (56) |
| *per^T^;Sp/CyO; cry^03^* | 19 (83) | 16 (+1/-0.3) | 4 (17) | 17 (94) | 16.3 (+0.7/-0.8) | 1 (6) | 18 (86) | 16 (+0.7/-1) | 3 (6) |
| *per^T^;Sp/CyO; cry^b^* | 12 (67) | 16.5 (+0.7/-1) | 6 (33) | 9 (82) | 16.5 (+0.8/-0.5) | 2 (18) | 8 (62) | 17.15 (+0.35/-0.65) | 5 (38) |
| *per^T^;Sp/CyO; cry^m^* | 15 (79) | 16.5 (+0.5/-1.3) | 4 (21) | 21 (91) | 16.5 (+1/-0.5) | 2 (9) | 14 (93) | 16.4 (+0.9/-0.4) | 1 (7) |
| *per^T^;Sp/CyO; Sb/TM6B* | 15 (75) | 16.5 (+0.5/-1) | 5 (25) | 17 (100) | 15.5 (+0.8/-0.5) | 0 (0) | 11 (85) | 15.7 (+1.3/-0.7) | 2 (15) |

| **Table S1. part C Free-running locomotion in DD at different temperatures** | | | | | | | | | |
| --- | --- | --- | --- | --- | --- | --- | --- | --- | --- |
| Genotype | 18°C | | | 25°C | | | 28°C | | |
|  | *N* rhythmic (%) | FRP (+/-) [hrs] | *N* non-rhythmic (%) | *N* rhythmic (%) | FRP (+/-) [hrs] | *N* non-rhythmic (%) | *N* rhythmic (%) | FRP (+/-) [hrs] | *N* non-rhythmic (%) |
| *y per^T^;Sp/CyO; cry^01^* | 20 (83) | 16.5 (+0.5/-0.5) | 4 (17) | 12 (67) | 16.15 (+1.15/-0.65) | 6 (33) | 11 (65) | 16 (+2/-1) | 6 (35) |
| *y per^T^;Sp/CyO; cry^02^* | 4 (18) | 16.85 (+0.65/-0.35) | 18 (81) | 11 (46) | 15.7 (+1.3/-0.4) | 13 (54) | 4 (29) | 16.75 (+0.3/-0.75) | 10 (71) |
| *y per^T^;Sp/CyO; cry^03^* | 11 (50) | 16.5 (+1/-0.8) | 11 (50) | 25 (86) | 16.7 (+1.3/-1.2) | 4 (14) | 10 (45) | 16 (+0.5/-1.5) | 12 (55) |
| *y per^T^;Sp/CyO; cry^b^* | 15 (65) | 16.3 (+1.2/-1) | 8 (35) | 19 (68) | 16.3 (+0.7/-1.3) | 9 (32) | 11 (55) | 16.5 (+1.5/-1.2) | 9 (45) |
| *y per^T^;Sp/CyO; cry^m^* | 16 (70) | 16.7 (+0.6/-1.4) | 7 (30) | 19 (90) | 16.7 (+0.3/-0.4) | 2 (10) | 23 (100) | 16.3 (+1/-0.6) | 0 (0) |
| *y per^T^;Sp/CyO; Sb/TM6B* | 16 (76) | 16.5 (+0.5/-1) | 5 (24) | 19 (76) | 16 (+1/-1) | 6 (24) | 7 (78) | 16.5 (+1/-0.5) | 2 (22) |
| *per^S^;Sp/CyO; cry^01^* | 21 (84) | 20 (+0.5/-1) | 4 (16) | 14 (67) | 19.15 (+1.35/-0.65) | 7 (33) | 13 (93) | 18.7 (+0.6/-0.4) | 1 (7) |
| *per^S^;Sp/CyO; cry^02^* | 5 (25) | 19 (+0.3/0.5) | 15 (75) | 12 (57) | 19.25 (+1.25/-1.25) | 9 (43) | 13 (72) | 18.85 (+0.65/-0.85) | 5 (28) |
| *per^S^;Sp/CyO; cry^03^* | 12 (60) | 19.5 (+0.5/-0.5) | 8 (40) | 19 (86) | 19 (+2/-0.3) | 3 (14) | 13 (87) | 18.8 (+0.9/-0.8) | 2 (13) |
| *per^S^;Sp/CyO; cry^b^* | 12 (67) | 19.5 (+0.5/-1) | 6 (33) | 2 |  |  | 6 | 18.7 (+1/0.4) | 0 |
| *per^S^;Sp/CyO; cry^m^* | 21 (91) | 19.5 (+1.5/-1.2) | 2 (9) | 25 (100) | 19.3 (+0.7/-0.3) | 0 (0) | 21 (100) | 19 (+0.7/-1) | 0 (0) |
| *per^S^;Sp/CyO; Sb/TM6B* | 13 (93) | 19.8 (+0.2/-1.1) | 1 (7) | 18 (86) | 18.5 (+0.5/-0.5) | 3 (14) | 15 (94) | 18.5 (+0.5/-0.5) | 1 (6) |

| **Table S1. part D Free-running locomotion in DD at different temperatures** | | | | | | | | | |
| --- | --- | --- | --- | --- | --- | --- | --- | --- | --- |
| Genotype | 18°C | | | 25°C | | | 28°C | | |
|  | *N* rhythmic (%) | FRP (+/-) [hrs] | *N* non-rhythmic (%) | *N* rhythmic (%) | FRP (+/-) [hrs] | *N* non-rhythmic (%) | *N* rhythmic (%) | FRP (+/-) [hrs] | *N* non-rhythmic (%) |
| *per^L^ ;* *+/+;*  *cry^01^* | 5 | 27 (+0.5/-0.5) | 0 | 6 (38) | 29.5 (+0.5/-0.5) | 10 (62) | 5 | 29.5 (+0.5/-0.5) | 0 |
| *per^L^; +/+;*  *cry^02^* | 20 (83) | 27.5 (+0.5/-1.5) | 4 (17) | 17 (77) | 28 (+1/-0.5) | 5 (23) | 17 (89) | 29 (+0.5/-0.5) | 2 (11) |
| *per^L^; +/+;*  *cry^03^* | 22 (92) | 26.5 (+1.0/-0.5) | 2 (8) | 17 (74) | 29.5 (+0.5/-2) | 6 (26) | 17 (81) | 30 (+0.5/-0.5) | 4 (19) |
| *per^L^; +/+;*  *cry^b^* | 11 (79) | 27 (+0.5/-1.5) | 3 (21) | 14 (88) | 29 (+1/-0.5) | 2 (12) | 9 (82) | 29 (+1.5/-0.5) | 2 (18) |
| *per^L^; +/+;*  *cry^m^* | 16 (76) | 28 (+1/-1) | 5 (24) | 18 (75) | 30 (+2/-2.5) | 6 (25) | 14 (82) | 32 (+1/-1) | 3 (18) |
| *per^L^; +/+; Sb/TM6B* | 14 (70) | 27.25 (+0.75/-0.75) | 6 (30) | 15 (79) | 30.5 (+2.5/-2) | 4 (21) | 10 (100) | 30 (+1.5/-1) | 0 (0) |
| *Sp/Cyo;cry^01^* | 22 (100) | 23.5 (+1/-0.5) | 0 (0) | 20 (100) | 23.5 (+1/-0.5) | 0 (0) | 13 (100) | 23 (+1.5/-0) | 0 |
| *Sp/Cyo;cry^02^* | 16 (84) | 23.5 (+2/-0.5) | 3 (15) | 18 (100) | 23.75 (+0.75/-1.25) | 0 (0) | 13 (93) | 23 (+1.5/-0) | 1 (7) |
| *Sp/CyO;cry^03^* | 23 (100) | 24 (+0.5/-0.5) | 0 (0) | 24 (100) | 24 (+0.5/-0.5) | 0 (0) | 14 (100) | 23.5 (+0.5/-0.5) | 0 (0) |
| *Sp/CyO;cry^b^* | 12 (100) | 23.5 (+1/-0.5) | 0 | 9 (90) | 24 (+0.5/-1) | 1 (10) | 9 | 23.5 (+0.5/-0.5) | 0 |
| *Sp/CyO;cry^m^* | 21 (95) | 23.5 (+1/-0.5) | 1 (5) | 19 (100) | 24 (+0.5/-0.5) | 0 (0) | 12 (86) | 23.5 (+0.5/-0) | 2 (14) |
